# Composite Microgels with Dynamically Switchable Stiffness

**DOI:** 10.1002/smll.76505

**Published:** 2026-10-30

**Authors:** Jana T. Reh, Leonard Funke, Berna Özkale, Oliver Lieleg

**Affiliations:** ^1^ School of Engineering and Design, Department of Materials Engineering Technical University of Munich Garching Germany; ^2^ Center For Protein Assemblies (CPA) Technical University of Munich Garching Germany; ^3^ Munich Institute of Biomedical Engineering Technical University of Munich Garching Germany; ^4^ Microrobotic Bioengineering Lab (MRBL), Department of Electrical Engineering, School of Computation, Information and Technology Technical University of Munich Garching Germany; ^5^ Munich Institute of Robotics and Machine Intelligence Technical University of Munich Munich Germany

**Keywords:** alginate, cross‐linking, indentation, mechanical properties, mucin

## Abstract

Whereas macroscopic hydrogels with precisely controllable and dynamically adjustable properties do exist, the development of similar platforms at the microscale is much more challenging. Here, we introduce a novel composite microgel system comprising the two cytocompatible biopolymers alginate and mucin, which are functionalized to simultaneously enable covalent and ionic cross‐linking. As a consequence, the initial stiffness of the generated microgels can not only be tailored over a range from ∼350 Pa to ∼7 kPa, but it is also possible to first increase and then decrease the microgel stiffness on demand at any time point post‐production. Owing to the two distinct biopolymer components in the microgels, they can be enzymatically degraded and resist biofouling events by bacteria. With this set of properties, this microgel system should be highly suitable for versatile applications in bioengineering or mechanobiology.

## Graphical abstract

Composite microgels comprising alginate and mucin are produced, in which covalent and ionics cross‐links are combined such that the mechanical properties of the microgels can be tuned on demand at any time post‐production. In addition, the mucin component conveys bacteria‐repellent properties to the cytocompatible microgels.

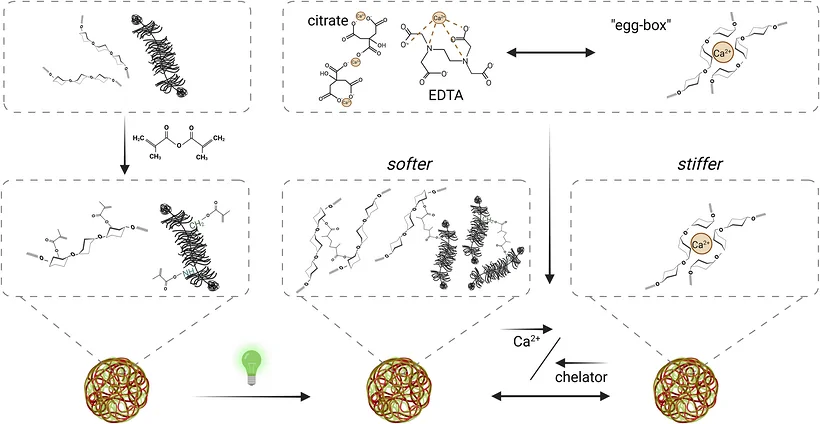

## 1 Introduction

Hydrogels are three‐dimensional (3D) networks of polymers that contain large amounts of water. This material class has been shown to be highly suitable for a range of different applications, e.g., as wound dressings [1], in the field of tissue engineering  [2], or as biosensors [3]. They can be fabricated from diverse synthetic or biological polymers, and the latter typically offer various advantages such as good biocompatibility and biodegradability [4]. By adjusting the concentration of (bio)polymers and cross‐links in such hydrogel systems, their properties can be tuned, enabling control over their viscoelasticity, mechanical stability, and degradation behavior. With lots of advances made in the field of macroscopic hydrogels [5], this material class is now further developed on the microscale by creating so‐called microgels [6, 7, 8, 9]. Microgels are stable, porous 3D structures that allow for nutrient penetration and offer the option to encapsulate drugs or cells. In comparison to commonly used two‐dimensional cell culture, such a 3D microenvironment comes with a range of advantages for cells [10].

To generate microgels, there are different methods such as emulsification [11], gas‐shearing [12], or microfluidics [13]. To date, microfluidics offers several advantages over the other two methods, as it allows for fine‐tuning the shape, size, and composition of the produced microgels by varying the chip geometry or the flow rate of the different liquid phases. In most setups, an oil phase shears off the polymer‐containing aqueous phase at a defined junction, resulting in the formation of polymeric spheres. To obtain stable, defined microgels, the polymer spheres then need to undergo cross‐linking, and this step can either be implemented into the fabrication process, or it can be conducted post‐production.

When working with biopolymer‐based microgels, one prominent method is ionic cross‐linking, e.g., Ca^2+^‐induced cross‐linking of the G‐blocks of alginate chains [14]. In addition to its ability to undergo this rapid and mild ionic cross‐linking process, alginate offers further advantages such as high abundance, low cost, and excellent cytocompatibility [15]. As a consequence, alginate‐based microgels are widely used in the field of bioengineering, e.g., as microrobots [16, 17], biosensors [18], or for cell activation [19, 20]. However, some applications require adjustable mechanical properties [21, 22]. To date, this is mainly achieved by constructing core‐shell systems  [23], or‐similar to macrogels‐ by varying the polymer [24, 25, 26, 27] and/or cross‐linker [28, 29] concentration and conditions (e.g., by adjusting the cross‐linking time  [30, 31]); the resulting mechanical properties are then static over time. Indeed, the development of systems with dynamically switchable mechanics has so far mostly been restricted to macroscale hydrogels [32, 33, 34].

Sometimes, a second (bio)polymer is introduced into alginate‐based microgels to further tune their properties, e.g., gelatin [35, 36]. However, owing to the tendency of mixed polymer systems to undergo phase separation, composite microgels typically do not exhibit a homogeneous mixture of their constituents [37], often require complex multi‐channel chips for their production [6, 38], or require pre‐cross‐linked, premixed polymer solutions.

With the resulting versatile options of material formulations, microgels can cover a broad range of mechanical properties suitable for diverse applications. Still, dynamically tuning the stiffness of these systems has not been achieved yet. Introducing such dynamic tunability might be highly beneficial for cell encapsulation approaches, as cells continuously sense the mechanical properties of their surroundings [39, 40]. By creating the option to adapt the stiffness of microgels on demand in a controllable, dynamic manner, it might be possible to guide the differentiation behavior of stem cells [29, 41].

One key objective of this work is to develop a novel microgel system whose mechanical properties can be tuned on demand. Those microgels are produced from a mixture of two cytocompatible biopolymers, the polysaccharide alginate and the glycoprotein mucin. This choice is motivated by results from a previous study [34], where we demonstrated the feasibility of dynamically switching the mechanical properties of a similar material formulation at the macroscale. Mucin is not only selected due to its ease of functionalization (which we here employ to conjugate methacrylate groups to enable covalent cross‐linking) but also for its anti‐biofouling properties [42], its ability to resist dehydration, and its immune‐modulating behavior [43, 44].

Here, we present mixed alginate/mucin microgels which are uniform, stable, and cytocompatible; moreover, the two biopolymer components are homogeneously distributed across the microgel volume. Depending on the formulation and the type of cross‐linking used, the initial microgel stiffness ranges from ∼350 Pa to ∼7 kPa; importantly, it is not only possible to switch the microgel stiffness up post‐production but also down again. To the best of our knowledge, such uniform composite microgels with dynamically switchable mechanics have not been developed yet. Additionally, the alginate component enables enzymatic degradation of thoroughly cross‐linked microgels, whereas the mucin component increases the biofouling resistance of the microgels toward bacteria. Taken together, those properties make alginate/mucin composite microgels promising candidates for diverse applications in the field of bio‐engineering and mechanobiology.

## 2 Results and Discussion

### 2.1 Material Characterization and Production of Microgels

To enable the formation of covalent cross‐links in microgels, the two biopolymers alginate and mucin are functionalized with methacrylic anhydride to obtain methacrylated alginate (mAlg) and methacrylated mucin (mMUC). Such covalent cross‐links between a given biopolymer variant or between each other (Figure 1a) should allow for the generation of stable microgels, whereas additional, transient cross‐linking with divalent ions should offer the possibility to conduct dynamic alterations in the microgel stiffness.

**FIGURE 1:**
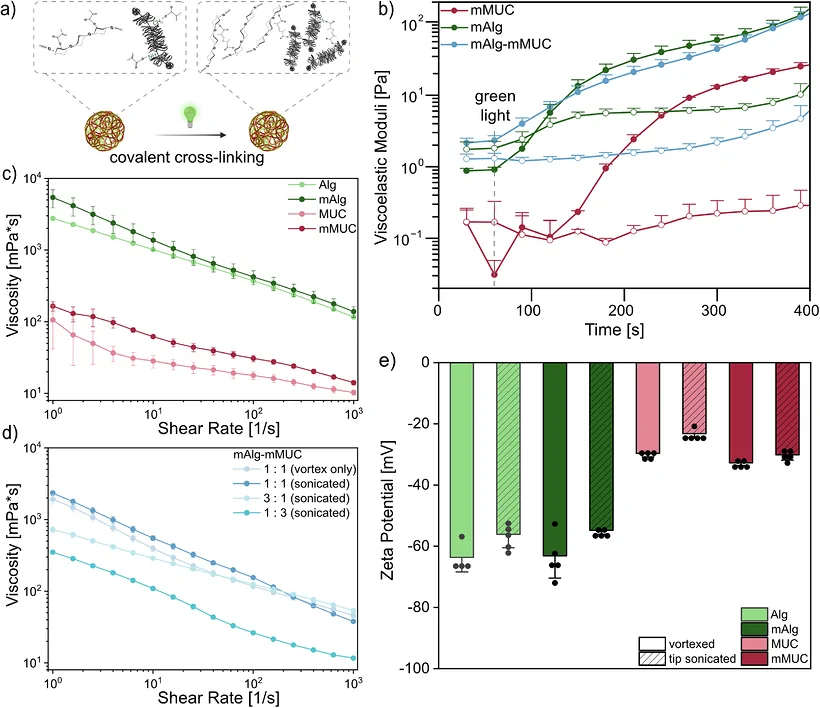
Rheological characterization and zeta potential of the biopolymer systems employed for microgel fabrication. (a) Illustration of the covalent cross‐linking of the methacrylate functionalized biopolymers mucin (MUC) and alginate (Alg) via illumination with green light. (b) Viscoelastic moduli G′ (closed symbols) and G″ (open symbols) of different biopolymer samples (mAlg, mMUC, mAlg‐mMUC 1:1) over time; illumination with green light is initiated after 1 min and induces gelation via covalent cross‐linking. (c,d) Viscosity of non‐cross‐linked biopolymer solutions (Alg, mAlg, MUC, and mMUC, and mixtures thereof) as a function of the shear rate. (e) Zeta potential of Alg, mAlg, MUC, and mMUC solutions which were either vortexed or tip sonicated. In all, the error bars denote the standard deviation. For rheological data, n = 3; for zeta potential data, n = 5. Figure 1a was made using BioRender: (2026) https://BioRender.com/pb02tbv.

To assess whether covalent cross‐linking is possible with these modified biopolymers, a macrorheological setup is selected which allows for creating light‐induced cross‐links in situ by illuminating a sample from below through a transparent plate. As a suitable photoinitiator system, a combination of the three molecules eosin‐y, triethanolamine (TEOA), and vinylpyrrolidone (VP) is selected based on findings by Etter et al. [45]. In detail, this photoinitiator system enables cross‐linking with green light, which allows for a broader range of possible applications compared to cross‐linking via UV light (which can be harmful for cells [46, 47]). Here, a slightly adjusted photoinitiator mixture indeed results in successful cross‐linking (Figure 1b)—not only of solutions containing one of the functionalized biopolymers, mAlg or mMUC, but also for mixed solutions (prepared at a mixture ratio of 1:1 and a total polymer concentration of 1% (w/v)). In the mixed system, it seems that mAlg dominates the covalent cross‐linking process: the resulting stiffness values are around 120 Pa after 5.5 min of illumination, and this is very similar to values obtained for the cross‐linked pure mAlg system prepared at the same total polymer concentration, but almost one order of magnitude higher than what we find for a cross‐linked mMUC system.

Having demonstrated that the prepared biopolymer conjugates can be cross‐linked well, we adapt a protocol for the microfluidic production of pure alginate gels and incorporate the second biopolymer, mucin, as well as the photoinitiator mixture into the material mix. Moreover, we add CaCO_3_ nanoparticles into the formulation to achieve a sufficient initial stability of the microgels during the collection process until further cross‐linking via light exposure can be conducted. Here, the release of Ca^2+^ ions from the CaCO_3_ nanoparticles is triggered in the microfluidic chip through direct contact of the produced microgels with the oil phase (which contains glacial acetic acid). To guarantee sufficient dissolution of the nanoparticles, the solvent of the biopolymer components was optimized to a custom‐made medium containing EDTA (DMEM‐EDTA‐HEPES). In conclusion, the aqueous phase used to produce the microgels contains a mixture of the two functionalized biopolymer components (mAlg and mMUC), the three components necessary for a photoinitiator‐driven cross‐linking reaction (see above), and the carbonate nanoparticles (Figure 2a).

**FIGURE 2:**
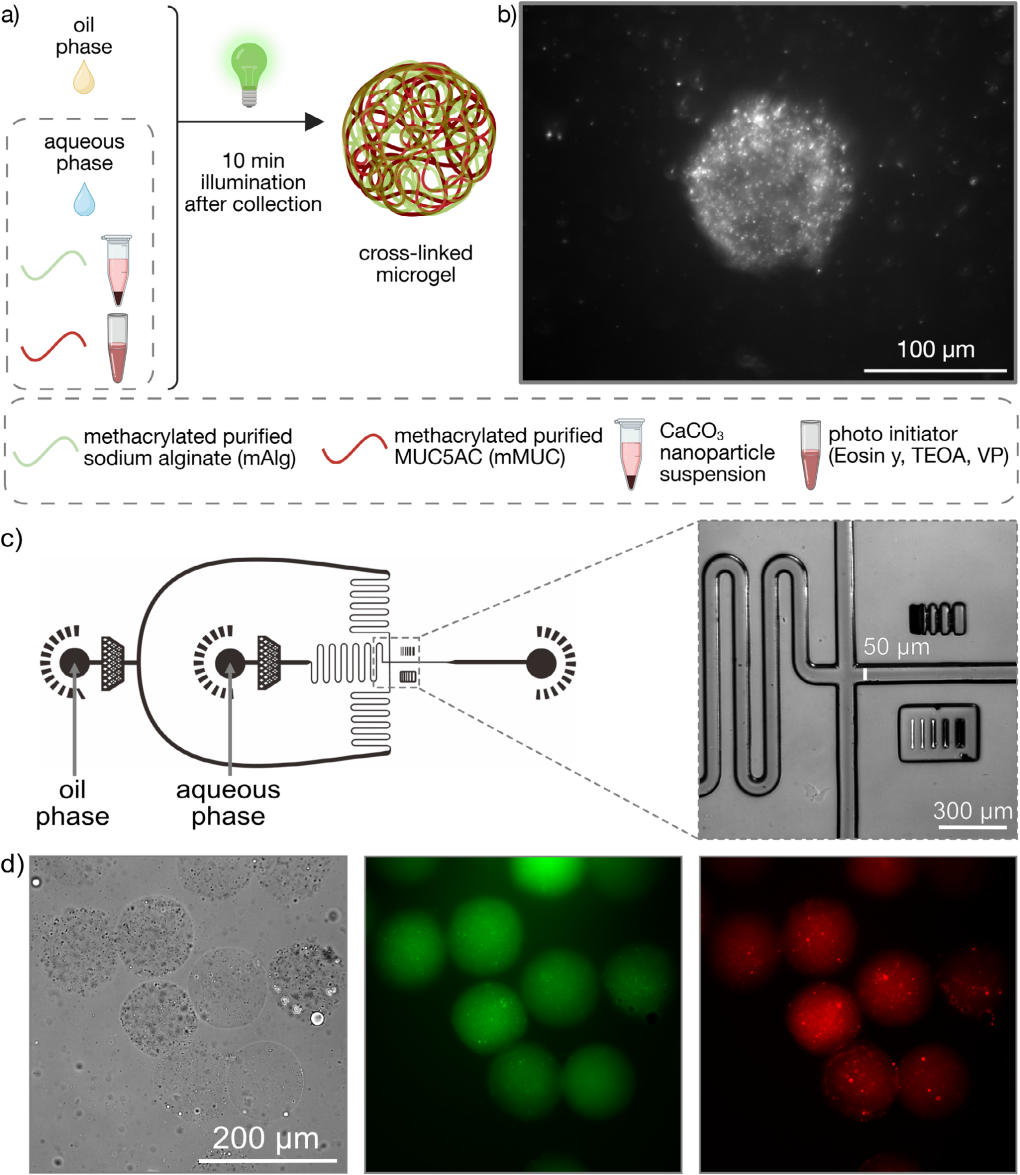
Production and morphology of composite microgels comprising mAlg and mMUC. (a) Schematic illustration of the components used in the microfluidic production process. The obtained microgels were covalently cross‐linked via illumination with green light for 10 min. (b) Representative darkfield image of a such produced microgel comprising a mAlg to mMUC ratio of 1:1. (c) Schematic illustration of the microfluidic chip with arrows indicating the inlets for the oil and the aqueous phase, respectively. A microscopic zoom‐in picture of the T‐junction, where the microgels are formed. Here, the channel size is 50 µm. (d) Representative microscopy images obtained in phase contrast mode (left) as well as in fluorescent mode (middle, right). The green channel depicts mAlg, whereas the red channel depicts mMUC. The scale bar in the phase contrast image represents 200 µm and applies to all images in (d). Figure 2a was made using BioRender: (2026) https://BioRender.com/24neuue.

To be able to reproducibly fabricate microgels from this complex mixture of components, it is first necessary to optimize the microfluidic process; here, especially phase separation is a major issue that needs to be tackled. Mixing the composite system by pipetting or vortexing alone leads to microgels of different sizes, the presence of large amounts of unshaped bulk material, or – in the best case – to inhomogeneous distributions of mAlg and mMUC within the microgel (see Figure S1). Such issues did not previously affect the viscoelastic properties of similar gels at the macroscale [34]; however, they now have a significant impact at the microscale. For instance, the flow in the microfluidic chip is sometimes interrupted during microgel fabrication (see Video S1), which is problematic.

To test if this problem arises from a too‐high viscosity of the biopolymer mixture unsuitable for a microfluidic application, we determine the viscosities of the non‐functionalized and methacrylated biopolymer solutions. We find that methyacrylation does not strongly affect the macroscopic viscosity of the different systems—neither for pure alginate or mucin solutions nor for their mixture (Figure 1c,d). Pure alginate microgels fabricated from similar polymer concentrations as employed here have already been reported in the literature [48, 49]; thus, since the viscosity values determined for our alginate/mucin composites are lower than those of pure alginate solutions prepared at similar total polymer concentrations, the mixed systems should be suitable for the production of microgels. Nevertheless, minor local fluctuations in the homogeneity of the mixed biopolymer solution could still be responsible for the interruptions in flow we observe on chip.

Importantly, this problem can be remedied by incorporating tip sonication into the preparation protocol of the aqueous phase: with this modification, we achieve a much higher homogeneity of the aqueous phase and, consequently, a continuous flow in the microfluidic setup (see Video S2). To ensure that this sonication treatment does not inflict substantial damage to the biopolymers, dynamic light scattering (DLS) measurements are employed to compare the zeta potential (Figure 1e) as well as the hydrodynamic diameter (see Figure S2a,b) of functionalized and unmodified biopolymers before and after tip sonication. Those control measurements only return minor changes after sonication treatment and indicate reduced biopolymer agglomeration (see Figure S2c). Moreover, the macroscopic viscosity of the mixed biopolymer solution is virtually identical when the solution is only vortexed or treated with tip sonication (Figure 1d). Thus, we implement tip sonication as a crucial step of the preparation protocol and obtain well‐shaped, round microgels (Figure 2b). To achieve this positive outcome, the flow rates of the two phases used for microgel production had to be adjusted. For the chip geometry used here (Figure 2c), optimal flow rates are 6 µL min^−1^ for the oil phase and 2 µL min^−1^ for the aqueous phase. Indeed, the microgels generated with those parameters are stable enough so they can be collected after ∼25 min to conduct light‐induced, covalent cross‐linking. Moreover, experiments conducted with fluorescently labelled biopolymers show that both biopolymers are homogeneously distributed throughout the generated microgels (Figure 2d).

In a next step, the biopolymer composition of the microgels is varied by altering the alginate‐to‐mucin ratio. As shown in Figure 1d, both a 3:1 mixture as well as a 1:3 mixture have a lower viscosity than the 1:1 system studied so far. Thus, they should in principle be compatible with the same microfluidic chip used to produce the 1:1 microgels. However, we find that using a higher relative mMUC content (1:3 mixture) results in the production of only few microgels (see Figure S3a). This low efficiency is likely due to the lower cross‐linking efficiency of the mucin biopolymer compared to mAlg (see above). Owing to this unsatisfactory outcome, mAlg‐mMUC mixtures with a ratio of 1:3 are not considered further. In contrast, the other two biopolymer ratios investigated here, 1:1 and 3:1, allow for the efficient production of spherical microgels (Figure 3a,d). For each microgel production batch, small spherical particles are visible in the microscope images; those constitute residual oil droplets, which remain in the sample after the centrifugation and cleaning process.

**FIGURE 3:**
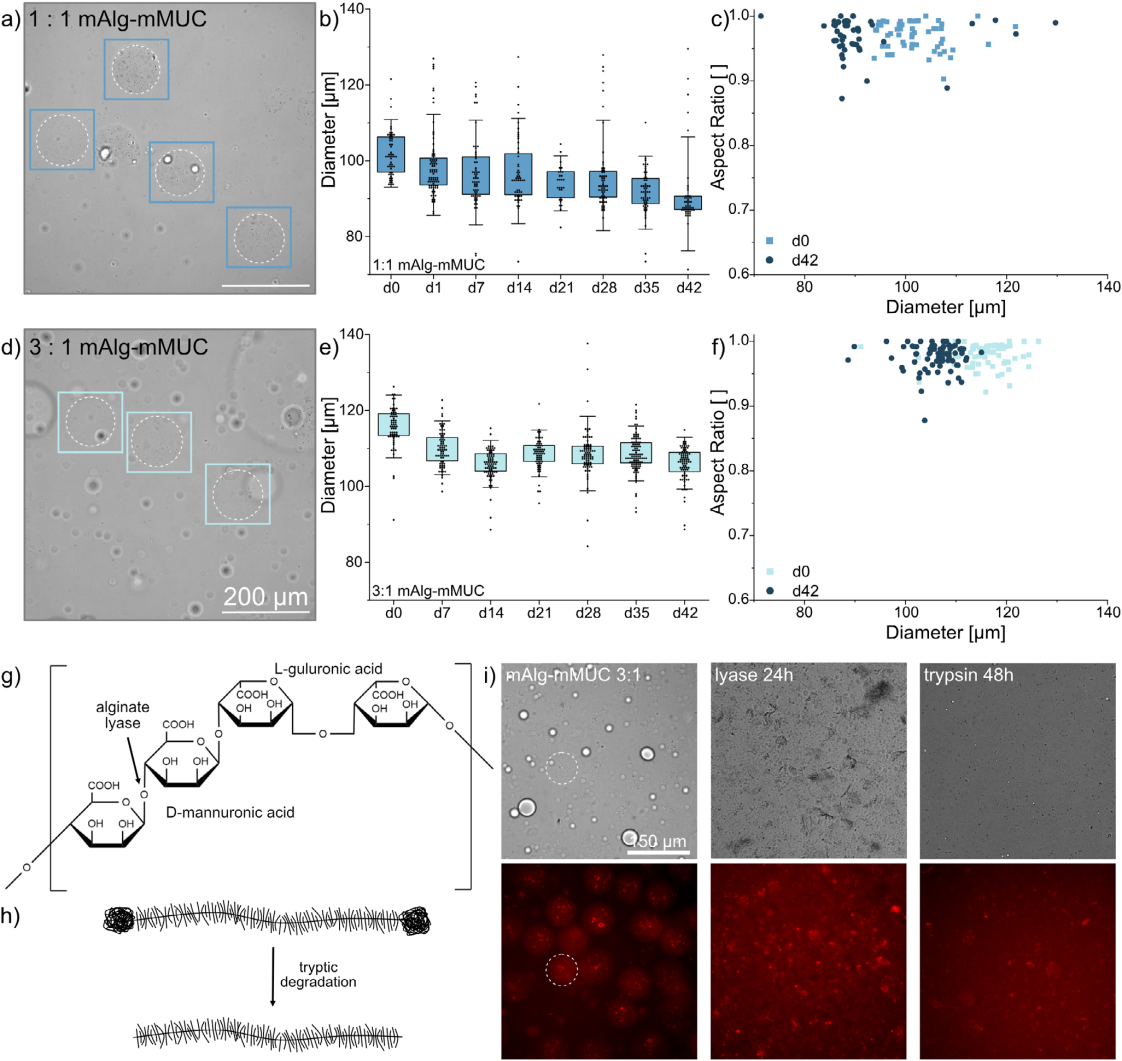
Size and shape analysis of mixed mAlg‐mMUC microgels over time. Microgels were produced at an mAlg to mMUC ratio of (a–c) 1:1 and (d–f) 3:1, respectively, and then were stored in ddH_2_O at 4°C for 42 days. During this time, the average diameter of the particles (b,e) as well as their aspect ratio (c,f) were determined. At each time point, 10 images were taken and all microgels in those images were analyzed. In (a,d) the scale bar represents 200 µm. In (b,e), one data point presents the average of three diameter measurements conducted per microgel. Error bars depict the standard deviation. (g) Schematic illustration of the chemical structure of D‐mannuronic and L‐guluronic acid, which are the two constituents of the alginate polymer. The bond where alginate lyase is expected to cut the biopolymer chain is indicated with an arrow. (h) Representative illustration of the reported degradation behavior of MUC5AC by trypsin, where primarily the mucin termini are cleaved. (i) Representative microscope images (brightfield and fluorescence) of mAlg‐mMUC 3:1 microgels. Untreated microgels are compared to lyase treated ones (treatment time: 24 h) and trypsin treated ones (treatment time: 48 h). The scale bar represents 150 µm and applies to all images in (i).

### 2.2 Stability Evaluation and Enzymatic Degradation

For microgels produced from the 1:1 formulation, we find average particle diameters in the range of 100 µm. When stored in D.I. water at 4°C, this value slightly decreases over time to ∼90 µm after 42 days (Figure 3b). However, during the same time interval, the aspect ratio of the particles remains nearly constant and close to 1 (Figure 3c), indicating a stable, spherical shape of the microgels. Microgels fabricated from the 3:1 mixture exhibit a slightly larger initial diameter of ∼115 µm, and their average size also slightly decreases over time, i.e., to ∼105 µm after 42 days (Figure 3e); also those 3:1 microgels maintain their spherical shape very well (Figure 3f). However, we find narrower size distributions for those microgels than for their 1:1 counterparts. We attribute this result to the higher relative mAlg content in this mixture, which should allow for improved cross‐linking (see Figure 1b), thus resulting in microgels with better‐defined average sizes.

For some applications such as cell encapsulation, it would be beneficial if the microgels could be degraded on demand (e.g., to release encapsulated cells again). However, as the introduced covalent bonds are not reversible, enzymatic degradation was assessed. Since the mixed microgels contain two different biopolymers, mAlg and mMUC, two different enzymes are tested: lyase and trypsin. The lyase used here targets the mannuronic block of Alg (see Figure 3g); in contrast, trypsin can cleave bonds between the amino acids L‐arginine and L‐lysine [50], which can be found at multiple locations in the peptide backbone of the mucin glycoprotein [51]. However, as the central region of mucin is strongly glycosylated, this area is well‐protected from enzymatic degradation; thus, an exposure of mucin to trypsin will mostly induce the cleavage of the mucin termini [52] (see Figure 3h), and it is unclear if this is sufficient to break down strongly cross‐linked mAlg/mMUC microgels. Indeed, when treating mAlg‐mMUC 3:1 microgels with trypsin at room temperature, small material aggregates are still visible after 48 h of incubation (see Figure 3i; for more time points, see Figure S5). Probably, this result can be explained by the fact that the methacrylate cross‐links are mainly generated between the glycosylated central regions of mucins, which are not affected by the enzyme. In contrast, incubating the same microgels with lyase at room temperature leads to fast degradation, and no visible microgels are left after 24 h of treatment (see Figure 3i; for more time points, see Figure S5).

### 2.3 Microindentation Measurements

Having demonstrated a successful production of mixed mAlg‐mMUC microgels with well‐defined sizes and spherical shape, we proceed to investigate the mechanical properties of these composite microgels by conducting microindentation measurements (Figure 4a). For this purpose, the microgels of interest are immobilized at the bottom of the wells of a 96‐well plate using a dopamine coating. Before adding and removing ionic cross‐links by supplying Ca^2+^ ions from the outside and removing them again with chelating agents, we evaluate different strategies to control the initial stiffness of those microgels: altering the mAlg to mMUC ratio, adding a poly‐*L*‐lysine (PLL) coating (Figure 4b), increasing the methacrylation degree on both biopolymer components, and increasing the total biopolymer concentration in the microgels. Please note that, in all those cases, the microgels exhibit a low degree of initial internal ionic cross‐linking (which results from the dissolution of the integrated CaCO_3_ nanoparticles) as well as covalent cross‐links created by illumination with green light.

**FIGURE 4:**
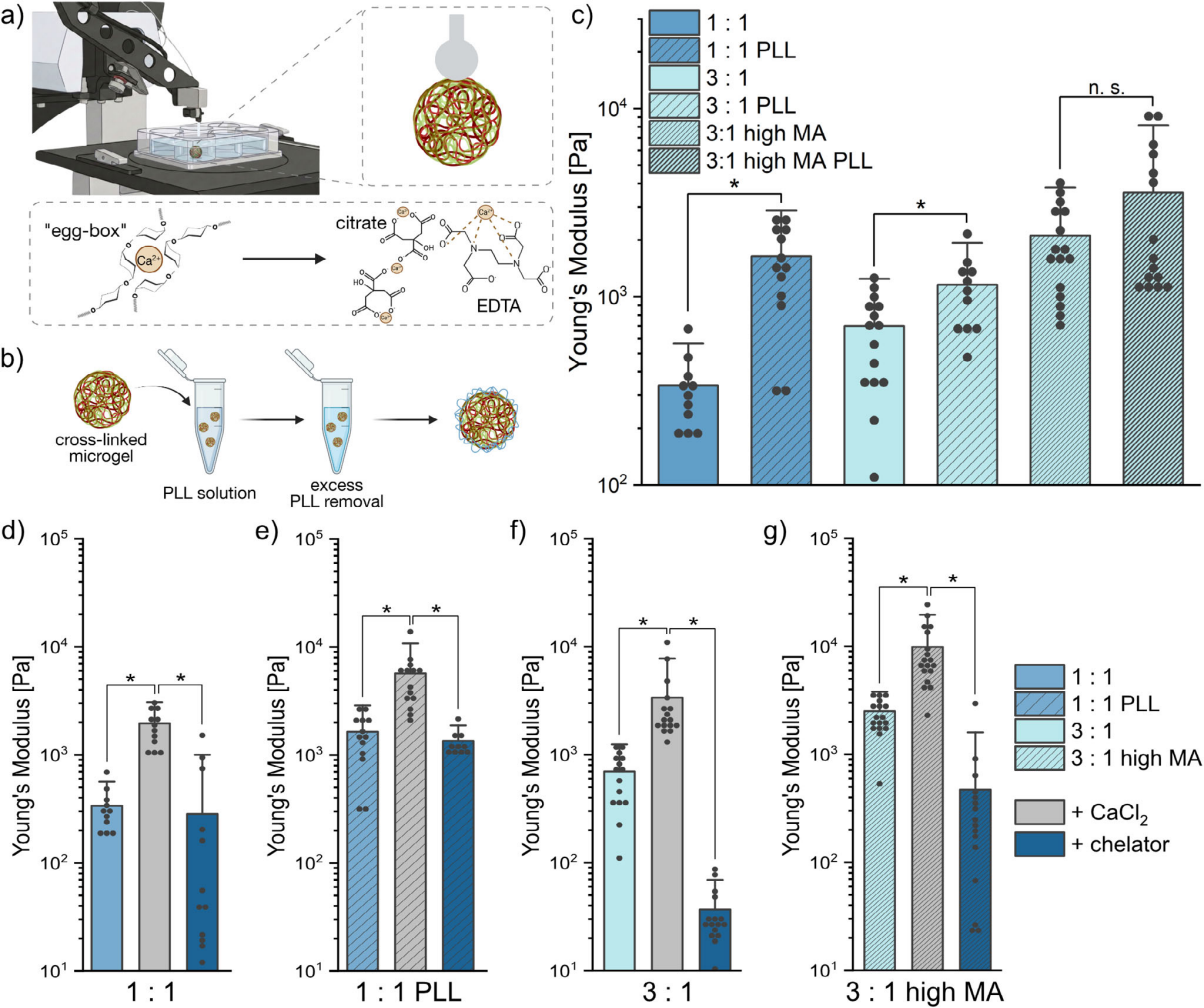
Stiffness of the microgels as reported by microindentation measurements. (a) Illustration of the setup used for the indentation tests. The microgels are immobilized to the bottom of the wells by a dopamine coating. The microgel stiffness is then dynamically tuned in situ by first adding CaCl_2_ (which induces cross‐linking of the alginate chains by forming a so‐called “egg‐box” structure) and then adding a mix of chelators (EDTA and citrate), which removes the ionic cross‐links again. (b) Schematic illustration of the process used to generate a PLL coating on the microgels. (c) Initial Young's moduli of different microgels right after their production. (d–g) Young's moduli of a subset of the microgel samples shown in (c) in three different states: right after production (left), after inducing additional ionic cross‐links by adding CaCl_2_ (middle), and after removing the ionic cross‐links by adding a chelator mixture (right). In (c–g), each symbol represents measurements from a distinct microgel, where the average from several consecutive indentations is shown. Error bars denote the standard deviation as determined from the tested microgel cohort. Asterisks denote statistically significant differences based on a p‐value of p = 0.05. Figure 4a,b was made using BioRender: (2026) https://BioRender.com/cxaz2xg.

As shown in Figure 4c, mAlg‐mMUC microgels fabricated from the 1:1 mixture exhibit a compressive stiffness of ∼300 Pa. With an additional PLL coating, this value is increased to ∼1.7 kPa; this result underscores that the PLL coating strategy works well, which was to be expected since the microgels comprise anionic macromolecules (Figure 1e). Owing to the positive net charge of PLL, it can engage in electrostatic interactions with the two anionic biopolymers. However, this PLL coating can be expected to be limited to a small surface layer due to the high molecular weight of PLL as well as of the biopolymers MUC and alginate, which leads to low chain mobility, thus limiting the diffusion of PLL into the microgel [19]. For mAlg‐mMUC microgels fabricated from the 3:1 mixture, the detected stiffness is ∼800 Pa. This increased value (compared to uncoated microgels fabricated from the 1:1 mixture) agrees with the higher mAlg content in these microgels, which allows for more efficient (covalent and ionic) cross‐linking. Also here, adding a PLL coating increases the compressive stiffness, this time to ∼1.2 kPa. Since this value is somewhat lower than what we obtain for the PLL‐coated 1:1 microgels, this suggests that PLL adsorption to the microgel surface is more efficient when more mucins are present. Nevertheless, the PLL coating can easily be adjusted by increasing the incubation time of the microgels in the PLL solution, and this leads to a strong increase in the average microgel stiffness up to ∼7.2 kPa (see Figure S3b).

However, depending on the later application of the microgels, a high initial stiffness might be required without employing a PLL coating. To reach this goal, the functionalization of alginate and mucin with methacrylic anhydride (MA) is optimized, and the success of this optimization is verified via macrorheological measurements (see Figure S3c) as well as absorbance and NMR measurements (see Figure S6–S8). With the optimized methacrylation process, the DS increases strongly from ∼1.3 for mAlg to ∼23.9 for the mAlg high MA variant (see Figure S6). A similar trend is observed when calculating the DS for mucin: we find ∼2k for mMUC and ∼4k for mMUC high MA (see Figure S7). For those high MA variants of either biopolymer, DLS measurements show a similar behavior of the hydrodynamic diameter and zeta potential (see Figure S2d–f) as for the low MA variants used to so far. Moreover, in full agreement with the macrorheological result, indentation of the new (high MA) mAlg‐mMUC 3:1 microgel variant reveals a strong increase in the compressive stiffness to ∼2.4 kPa. With an additional PLL coating, this value can be further increased to ∼3.6 kPa. It is important to note that the improved cross‐linking effect observed for the high MA variants of the biopolymers is mainly inferred from the obtained differences in mechanical properties (since determining the internal degree of cross‐linking in the microgels was technically not feasible). Of course, other factors such as alterations in the local network architecture or supramolecular interactions brought about by the higher degree of methacrylation might also affect the mechanical properties of the microgel system.

Finally, we also test if a higher total biopolymer concentration of 2% (w/v) might lead to an increase in the microgel stiffness. However, the detected stiffness of such created microgels is not significantly higher than what we obtain for microgels with a lower total biopolymer content (see Figure S3d). This might be a result of the heterogeneous distribution of biopolymers at the surface of those 2% (w/v) microgels (see Figures S3e and S4): since the measuring tip of the indentation setup has only a radius of 10.5 µm, such surface irregularities are likely to affect the accuracy of the measurement.

Having shown that it is possible to produce microgels with a mixed alginate/mucin content such that their initial stiffness can be tuned from a few hundred Pascal to ∼7.2 kPa, we next aim at dynamically modulating the mechanical properties of the microgels. Therefore, the previously characterized microgel variants are incubated with a CaCl_2_ solution (at a final concentration of ∼16 mM CaCl_2_), and the resulting increase in the microgel stiffness is quantified. For the 1:1 mixture, this leads to an increase in compressive stiffness by almost one order of magnitude, i.e., from ∼300 Pa to ∼2.2 kPa (Figure 4d). (Please note that the data shown was acquired after incubating the microgels with the CaCl_2_ solution overnight; when indentation measurements are conducted directly after the addition of CaCl_2_, a broader distribution of values is obtained.) This Ca^2+^‐induced increase in stiffness can be fully reverted by adding a suitable mixture of chelators (EDTA and citrate, see Experimental Section). Importantly, upon chelator addition, the microgels only soften but do not dissolve—and this stability is a result of the covalent cross‐links generated between the methacrylated biopolymers. We emphasize that only a very small volume of the chelating solution (corresponding to final concentrations of ∼0.6 mM EDTA and ∼0.3 mM citrate) is necessary to achieve a strong change in stiffness within minutes after addition. Moreover, some of the microgels are softer after chelator addition than in their initial state; this can be explained by the fact that, as a result of CaCO_3_ dissolution, all microgels already contain a small amount of ionic cross‐links before the CaCl_2_ solution is added—and also those initial ionic cross‐links can be removed by the chelating agents.

As the cationic PLL layer added to the surface of the microgels exhibits a different charge state than the two anionic biopolymers present in the core of the microgels (alginate and mucin are both anionic at neutral pH), it is important to test if the PLL coating interferes with triggering a dynamic change in mechanical properties. For instance, a cationic surface could reduce the efficiency of Ca^2+^ penetration into the microgels by establishing repulsive electrostatic forces, or it might trap the anionic chelators in the surface shell of the microgels by electrostatic attraction. However, upon addition of CaCl_2_ to 1:1 microgels carrying a PLL coating, we still find a strong increase in the microgel stiffness from ∼1.7 kPa to ∼6.2 kPa. Similarly, when introducing the chelating agent into the system, a strong softening to ∼1.5 kPa is observed (Figure 4e). This demonstrates that dynamically tuning the stiffness of the microgels is still possible if a PLL coating is employed. However, we note that a higher concentration of the chelating agents (∼1.6 mM EDTA and ∼0.8 mM citrate) is required now to achieve the described changes in compressive stiffness.

For microgels fabricated from the 3:1 mixture (where the relative mAlg content is higher), dynamically switching the microgel stiffness works very well, too. Here, upon the addition of Ca^2+^ ions, the stiffness of the microgels is increased from ∼800 Pa to ∼3.5 kPa (Figure 4f), and adding the chelator mixture induces softening to ∼40 Pa. We attribute this stronger decrease in stiffness induced by the chelating agents to the higher mAlg content in these microgels: the higher amount of mAlg chains is likely to allow for stronger ionic cross‐linking by Ca^2+^ ions—not only during microgel exposure to the CaCl_2_ solution but also during microgel production when Ca^2+^ is released from the CaCO_3_ particles. Accordingly, adding the chelator mixture has a stronger effect on this particular microgel variant than on those fabricated from the 1:1 mixture. This process can be repeated over several cycles with consistent outcomes (see Figure S9). Finally, for the microgels fabricated from the high MA variants of the two biopolymers, the microgel stiffness can be switched up to ∼10 kPa upon CaCl_2_ addition and back down to ∼600 Pa upon chelator addition (Figure 4g). Taken together, the indentation measurements nicely demonstrate the ability to dynamically and reversibly switch the stiffness of the mAlg‐mMUC microgels.

### 2.4 Cytocompatibility and Anti‐Biofouling Tests

For such microgels to be usable in combination with living cells (e.g., for cell encapsulation or as building blocks to generate ‘proto‐tissues’, where cells cross‐link the microgels into larger, 3D assemblies), a good cytocompatibility is of utter importance. For the formulations used here, the two biopolymers alginate and mucin should be unproblematic, since both macromolecules have been shown to not induce cytotoxic effects in prior studies [34]. However, the photoinitiator mixture can be cytotoxic if not fully consumed by the light‐driven cross‐linking reaction [34]. Thus, we here test this point by employing NIH/3T3 fibroblast as a model cell line.

mAlg‐mMUC microgels (at mixture ratios of 1:1 and 3:1) are produced and used to create a ‘leaching medium’. This leaching medium then should contain (putative) degradation products or unreacted components (such as residual photoinitiator molecules) which have left the microgels via diffusion. As another experimental group, pure alginate microgels (employing non‐functionalized biopolymers) exclusively stabilized by ionic cross‐links are tested as well. When fibroblasts are incubated with such leaching media overnight, we do not detect any signs of cytotoxic activity (Figure 5a). To ensure that this positive result does not originate from the low amount of microgel material present when generating the leaching medium, this experiment is repeated by using bulk hydrogels for leaching medium generation, and we obtain comparable results (Figure 5b). In other words, all material formulations tested here return cell viability values similar to control conditions, where cells are incubated with fresh medium only (Figure 5c). Moreover, viability experiments conducted for samples with a total biopolymer concentration of 2% (w/v) as well as for samples prepared from the high MA biopolymer versions did not return any indications of cytotoxic effects either (see Figure S10a–c).

**FIGURE 5:**
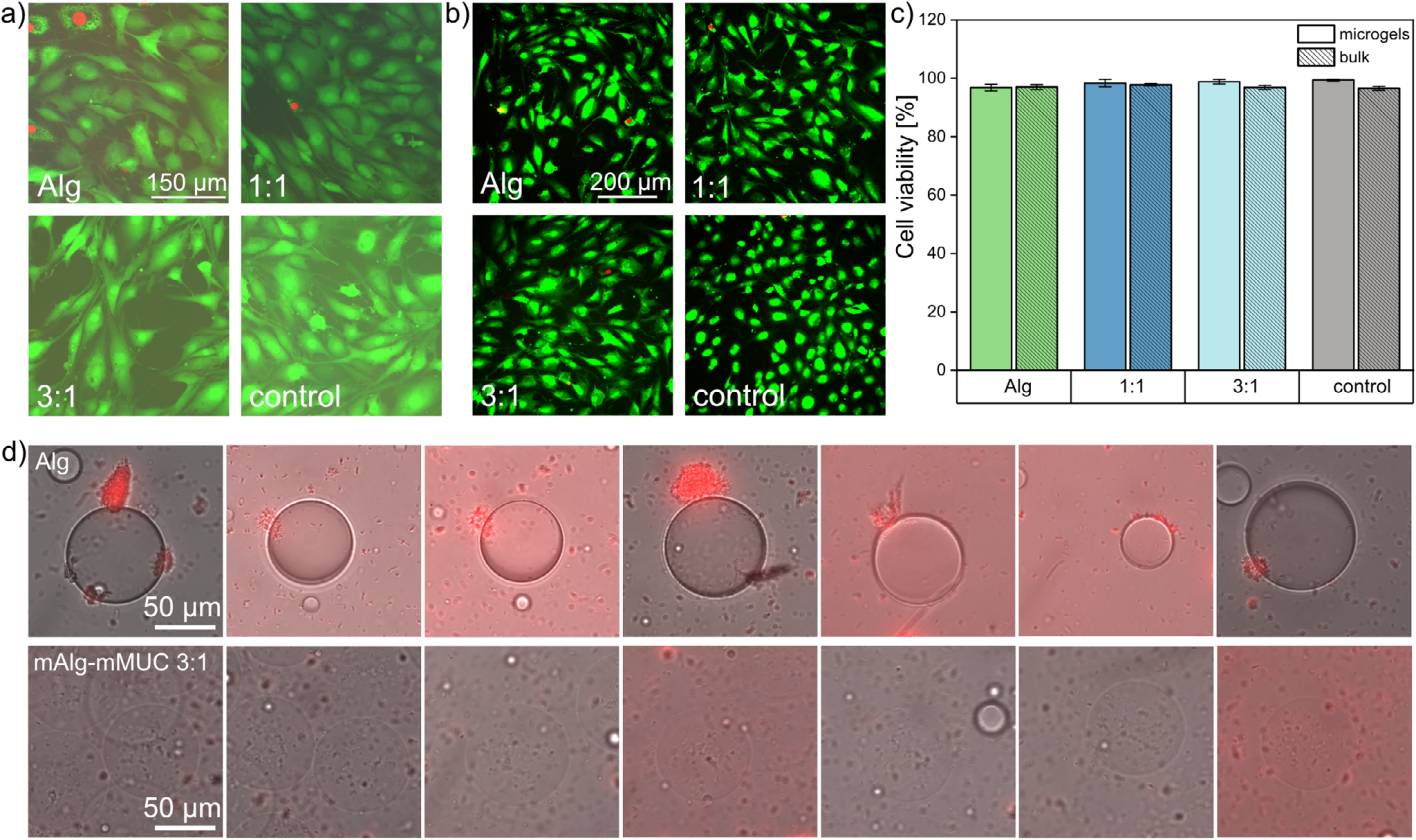
Cytocompatibility analysis of mixed alginate/mucin gels. Exemplary microscope images showing results of live/dead staining tests conducted with (a) microgels and (b) bulk gels, each prepared at a biopolymer mixture ratio of 1:1 and 3:1 and a total biopolymer concentration of 1% (w/v). The scale bars represent 150 and 200 µm, respectively, and apply to all images in the corresponding subfigure. (c) Cell viability values as calculated from the images shown in (a,b) as well as similar ones. Cells incubated with medium only were used as a control group. (d) Exemplary microscope images of a biofouling experiment investigating the adhesion of bacteria to the microgels. The scale bar represents 50 µm and applies to all images in this subfigure.

Of course, in a real biomedical application, cells would be in direct contact with the microgels, and such a scenario might return a different viability outcome. However, when we directly incubate fibroblasts with microgels, we do not find indications for cytotoxic effects either (see Figure S10d).

Finally, since the introduction of the glycoprotein mucin into the microgels was partially motivated by its anti‐biofouling properties documented in a range of macroscopic assays [34, 53], we test if this anti‐biofouling effect is also perceivable at the microscale. To do so, microgels were incubated in a solution of mCherry labeled *E. coli* bacteria for 1 week. As shown in Figure 5d, those fluorescent bacteria can attach to the surface of microgels prepared from pure alginate, where they then grow into small biofilm‐like clusters. Such biofilm formation is a microbiological event that comes with a range of problematic consequences, including increased antibiotic tolerance and resistance, and the latter is crucial to avoid in biomedical settings  [54, 55]. In contrast, for microgels containing only 25% mucin (mAlg‐mMUC 3:1), we do not find such bacteria clusters on the microgel surface. Consistently, also for higher concentrations of mMUC present in mAlg‐mMUC 1:1 microgels (see Figure S10e), mMUC conveys anti‐biofouling properties. In other words, introducing mucin into the material formulation indeed efficiently prevents bacterial adhesion to the microgels.

## 3 Conclusions

Here, we present composite microgels, which were fabricated from functionalized versions of the two biopolymers alginate and mucin and were designed to exhibit dynamically switchable stiffness. Whereas current microgel systems already offer a broad range of mechanical properties, they do not allow for tuning those properties post‐production. In contrast, the stiffness of the microgels described here can be switched up and down again in situ—whenever desired. Such mixed alginate/mucin microgels are stabilized by a combination of covalent and ionic cross‐links and do not only exhibit a homogeneous material distribution but also long‐term stability. Importantly, the covalent cross‐links do not inhibit enzymatic degradation of the alginate matrix, which allows for a putative release of cargo/encapsulated cells on demand. Moreover, owing to the mucin component in the microgels, we find improved anti‐biofouling properties, which will facilitate conducting long‐term experiments with this microgel platform.

This work represents a first approach to generate dynamic 3D microenvironments and lays the groundwork for future use of the microgels, *i.a*. for controlling the differentiation pathway of stem cells. We envision that such adaptable mechanical properties will enable improved control over the fate of stem cells synergistically with biomolecular signaling, and this could potentially open new opportunities for stem cell therapy.

## 4 Experimental Section

### 4.1 Chemicals

Unless stated otherwise, all chemicals used in this study were purchased from Carl Roth GmbH & Co. KG (Karlsruhe, Germany).

### 4.2 Mucin Purification

MUC5AC, which is the mucin variant used in this study to generate microgels, was obtained from porcine gastric tissue and was manually purified. MUC5AC purification was performed according to a previously published protocol [56] with minor changes. In brief, raw mucus was collected by carefully scraping the inner tissue surface of fresh, rinsed pig stomachs and afterwards diluted 5‐fold in 10 mM PBS (pH = 7.0) containing 170 mM sodium chloride (NaCl) and 0.04% sodium azide. This mixture was further stirred at 4°C overnight and subsequently treated by filtration using metal filter grids with three different grid sizes (500, 200, and 150 µm) and an ultracentrifugation step (150 000 g at 4°C for 1 h) to remove cellular debris. Afterwards, size exclusion chromatography was carried out using an ÄKTA purifier system (GE Healthcare, Munich, Germany) equipped with a XK50/100 column packed with Sepharose 6FF (GE Healthcare). Furthermore, the mucin‐containing fractions were collected, and the NaCl concentration was increased to 1 M. Subsequently, the mucin solution was concentrated with the help of a crossflow filtration unit (Xampler Ultrafiltration Cartridge, GE Healthcare; MWCO: 100 kDa) and finally lyophilized to allow storage until further use at −80°C.

### 4.3 Alginate Purification

To purify alginate, the following protocol was employed. First, 1 g of high molecular weight alginate (I‐1G, 280 kDa, Kimica, Tokyo, Japan) was dissolved in 100 mL ddH_2_O and fully dissolved overnight. Afterwards, 0.5 g of activated charcoal (Sigma‐Aldrich) was added per gram of alginate and stirred for 30 min. The activated charcoal was allowed to settle; then, the mixture was filtered using a 0.22 µm filter. Subsequently, the purified material was lyophilized and stored at −80°C until use.

### 4.4 Methacrylation of Mucin and Alginate

To enable covalent cross‐linking using green light, both biopolymers used in this project, alginate and mucin, were functionalized with methacryl groups to obtain methacrylated alginate (mAlg) and methycrylated mucin (mMUC), respectively. For this purpose, 1% (w/v) of either macromolecule was dissolved in ultrapure water. While continuously stirring the solutions, they were cooled down to 4°C, and the pH was adjusted to 8.0 by using 1 M sodium hydroxide (NaOH). Methacrylic anhydride (Sigma‐Aldrich) was then added in a ratio of 8.47 × 10^−4^ mL per mg of biopolymer. The pH was further maintained at 8.0 for 24 h. Subsequently, the solution was centrifuged and then dialyzed using a semipermeable membrane (12–14 kDa, Spectrum, Spectra/Por, Spectrum Laboratories, Inc., USA) in ultrapure water for a minimum of 5 days. During dialysis, the water was exchanged every day. Afterwards, the biopolymer solution was lyophilized and stored at −80°C until further use.

In a second step, this process was optimized to achieve more efficient cross‐linking. Therefore, 7 × 10^−3^ mL of methacrylic anhydride were slowly added per mg of biopolymer solution.

### 4.5 Photoinitiator

A mixture of eosin‐y, triethanolamine (TEOA), and 1‐vinyl‐2‐pyrrolidone (VP, Sigma‐Aldrich) was prepared in ddH_2_O employed as photoinitiator used to generate covalent cross‐links upon illumination with green light (5 m LED, 532 nm, 3200 lm m^−1^, Novectro GmbH & Co KG, Hörbranz, Austria). For each sample, 10% of the volume was constituted by this photoinitiator mixture, with final concentrations of 0.1 mM eosin‐y, 125 mM TEOA, and 20 mM VP [45, 57].

### 4.6 CaCO_3_ Nanoparticle Preparation

For preparing a CaCO_3_ nanoparticle (CalEssence, PCC70, Minerals Technologies, New York, United States) solution, the nanoparticles were first sterilized via autoclaving at 121°C for 20 min. Then, ∼15 mg sterile particles were weighed into a 1.5 mL microcentrifuge tube and suspended in 600 µL of Dulbecco's Modified Eagle Medium (DMEM, Sigma‐Aldrich). Subsequently, the suspension was sonicated for 15 s at 70% amplitude (Fisherbrand Model 120 Sonic Dismembrator, Thermo Fisher Scientific, Massachusetts, United States) and further diluted with 23 mL DMEM. This mixture was then centrifuged at 50 rcf and 20°C for 5 min, then the top 20 mL were removed and centrifuged again, this time at 1000 rcf for 5 min. Afterwards, the supernatant containing nanoparticles was removed, and the pellet was resuspended in complete DMEM to a concentration of 10 mg mL^−1^.

### 4.7 Medium Preparation

Medium was prepared by dissolving DMEM powder (high glucose, Gibco, Thermo Fisher Scientific) in ddH_2_O, adding 1% (v/v) FBS (heat‐inactivated FBS, Gibco, Thermo Fisher Scientific), 1% (v/v) penicillin/streptomycin (P/S), buffering the solution with 25 mM HEPES, and adjusting the pH to 7.8. 4% (w/v) CaCO_3_ nanoparticles (see above) were added, and the medium was stirred for 30 min before sterilizing it via filtration with a 0.22 mm filter (HEPES‐DMEM). To obtain EDTA‐HEPES‐DMEM, 1.8 mM of EDTA was added to chelate calcium ions. For complete DMEM, 10% (v/v) FBS, and 1% (v/v) P/S were added to Dulbecco's Eagle's high glucose medium (DMEM, Sigma‐Aldrich).

### 4.8 Rheological Measurements

To determine the viscosity of the polymer solutions of this study, a research‐grade shear rheometer (MCR 302, Anton Paar, Graz, Austria) was equipped with a cone‐plate geometry (CP25‐1 measuring head, and P‐PTD200/56 plate, Anton Paar). The measurements were conducted at 20°C over a shear rate range from 1 to 1000 s^−1^. For each sample type, three different samples were measured.

To determine the viscoelastic properties of samples over time (in response to cross‐linking via illumination with green light), the rheometer (Anton Paar) was equipped with a planar measuring head (PP25/Q1–79044, Anton Paar) and a transparent bottom plate (P‐PTD200/GL, Anton Paar). Illumination with green light was conducted from below using the same LED light described above. The measurements were carried out in torque‐controlled mode (*M* = 1 µNm) at 20°C using a gap size of 0.3 mm; data were recorded at a probing frequency of 1 Hz. For each sample type, three different samples were measured.

### 4.9 Zeta Potential Measurements

To investigate the zeta potential of the biopolymers and their functionalized forms, electrophoretic light scattering measurements were performed on a Litesizer 500 Zetasizer (Anton Paar), using the Smoluchowski approximation. For this purpose, 0.1% (w/v) of the material was dissolved in ddH_2_O. Then, the zeta potential was measured in an Omega cuvette (Anton Paar) at a target temperature of 25°C. Each biopolymer variant was characterized with *n* = 5.

### 4.10 Microfluidic Chip Preparation

Microfluidic chips were prepared by using a master, which was fabricated by conventional soft photolithography. Therefore, a thin layer (25 µm) of the photoresist SU8‐3050 (Kayaku Advanced Materials, Massachusetts, United States) was generated on a silicon wafer via spin coating (Spin150i, SPS‐International, Putten, Netherlands). Then, UV exposure with a dose of 250 µJ cm^−2^ was applied for 15 min with a tabletop maskless aligner system (µMLA, Heidelberg Instruments, Heidelberg, Germany) to locally cure the photoresist. Baking as well as development of the substrate were carried out for 5 min each. The wafer was then covered with a 10:1 mixture of polydimethylsiloxane (PDMS) and curing agent (Sylgard 184, Dow Corning Inc., Midland, United States), degassed for ∼2 h, and baked at 70°C for at least 2 h. The PDMS mold was subsequently removed, cut into shape, and holes were created using a biopsy punch (1 mm, pfm medical, Cologne, Germany). Such prepared mold was cleaned (together with a glass slide of suitable size) in 35% (v/v) ethanol in an ultrasonic bath for 5 min followed by rinsing with ultrapure water and a second ultrasonic bathing step in ultrapure water. This cleaning procedure was followed by a drying step in the oven at 65°C. The mold as well as the glass slide were then treated in a plasma oven (SmartPlasma 2.0, plasma technology, Herrenberg‐Gülstein, Germany) for 30 s by employing a pressure of 0.4 mbar at an intensity of 30 W and subsequently bonded together. The obtained chip was further cured in the oven at 80°C overnight. To create hydrophilic channels, the chip was flushed using RainX (Illinois Tool Works Inc., Krafft S.L.U., Andoain, Spain), which was afterwards evaporated in the oven at 65°C.

### 4.11 Microgel production

#### 4.11.1 Oil Phase

For preparing the oil phase, glacial acetic acid was diluted in ethyl 1,2,2,3,3,4,4,4‐octafluoro‐1‐[1,2,2,2‐tetrafluoro‐1‐(trifluoromethyl)ethyl]butyl ether (HFE, Novec 7500 engineered fluid, 3 M, Sigma‐Aldrich) to a final concentration of 1% (v/v). 46 µL of this diluted acid was then mixed with 1200 µL of a surfactant stock solution, which comprised 0.5% (v/v) PicoSurf (5% (w/w) in Novec 7500, Sphere Bio, Cambridge, UK) diluted in HFE. The oil phase was freshly prepared for each experiment.

#### 4.11.2 Aqueous Phase

To produce the aqueous phase, mAlg and mMUC were dissolved 2% (w/v) each in EDTA‐HEPES‐DMEM overnight. 240 µL of a polymer mixture containing a certain mass ratio (3:1, 1:1, or 1:3) of mMUC and mAlg was then mixed with 48 µL of the photoinitiator, 92 µL of EDTA‐HEPES‐DMEM, and 100 µL of the CaCO_3_ nanoparticle suspension (see above), which resulted in a final total biopolymer concentration of 1% (w/v). In addition, a higher total biopolymer concentration was tested as well, for which 4% (w/v) stock solutions of mAlg and mMUC were prepared and later diluted with photoinitiator, EDTA‐HEPES‐DMEM, and CaCO_3_ nanoparticle suspension to a final concentration of 2% (w/v). As a control group for anti‐biofouling and cytotoxicity tests, pure alginate microgels were prepared as well. In all cases, the final aqueous solution was sonicated for 15 s at 70% amplitude (Thermo Fisher Scientific).

#### 4.11.3 Microfluidic Setup

The microfluidic setup consisted of a 2‐channel chip with a T‐junction. The oil and aqueous phases were filled into 1 mL syringes (Omnifix‐F, B.Braun, Melsungen, Germany), which were connected via tubes (polytetrafluoroethylene (PTFE), ID 0.4 mm, Bohlender, Grünsfeld, Germany) and needles (Sterican, 27G, B. Braun) to the chip and to a syringe pump (LA100, Landgraf Laborsysteme, Langenhagen, Germany). Here, the flow rates were adjusted to values around 2 µL min^−1^ for the aqueous phase and 6 µL min^−1^ for the oil phase. The generated microgels were collected in a pipette tip (200 µL gel tip, VWR/ Avantor, Pennsylvania, United States) for about 25 min and then transferred to 1.5 mL Eppendorf tubes filled with 0.5 mL of deionized (D.I.) water. Afterwards, the tubes were illuminated with green light (see above) for 10 min to trigger covalent cross‐linking via the anhydride groups. For this purpose, the tube was placed into a glass beaker surrounded by LEDs (290 klx), and the glass beaker was placed onto a shaker.

After cross‐linking the samples with light illumination, the emulsion needed to be broken. This was achieved by adding ∼60 µL of 1H,1H,2H,2H‐perfluoro‐octyltriethoxysilane (PFO, Thermo Fisher Scientific) to each Eppendorf tube and slightly shaking it by turning it 10 times. Afterwards, the material was left to sediment, and the oil phase, which settled at the bottom of the tube, was removed and discarded. Then the tubes were centrifuged in a table centrifuge (Sprout, Sigma‐Aldrich), followed by another round of oil removal. This process was repeated until no separate oil phase was visible anymore.

### 4.12 Preparation and Imaging of Fluorescent Microgels

mMUC and mAlg were labeled with two distinct fluorophores, i.e., with ATTO 594 NHS‐ester (*E_x_/E_m_*: 603/626 nm, ATTO‐TEC GmbH, Siegen, Germany) and ATTO 488 NHS‐ester (*E_x_/E_m_*: 500/520 nm, ATTO‐TEC GmbH), respectively, and those labeled biopolymer variants were employed for microgel fabrication. In brief, 0.2% (w/v) of each biopolymer was dissolved in PBS buffer at pH 7. The solutions were then incubated with 1.65% (v/v) of the respective dye for 3 h and afterwards transferred into semipermeable membranes (300 kDa, Spectrum Laboratories) for dialysis. Dialysis was carried out for 2 days in D.I. water, which was changed three times. The conjugates were then lyophilized and stored at −80°C until further use. Please note, that the options for fluorescent labels are limited, as the required illumination with green light imposes restrictions. For imaging the labeled microgels, they were prepared at a total biopolymer concentration of 1% (w/v) of the biopolymers. Here, only 5% of the total biopolymer composition carried a fluorescent label. The final microgels were then imaged using a fluorescence microscope (DMi8, Leica, Wetzlar, Germany) employing a FITC (*E*_x_ = 460–500, DC = 505; *E*_m_ = 512–542, Leica) and a TXR filter cube (*E*_x_ = 540–580, DC = 585; *E*_m_ = 592–668, Leica), respectively.

The exemplary dark field image was obtained on an upright light microscope (Axioskop 2 plus, Zeiss, Oberkochen, Germany) using a 40× objective (LD Achroplan, 40× / 0.60 Korr Zeiss) and a digital camera (Orca‐R^2^, C10600, Hamamatsu, Japan).

### 4.13 Microgel Stability Evaluation

To assess the stability of the microgels, samples were incubated in ddH_2_O at either 4°C or 37°C. At different time points of sample storage, brightfield images were acquired on a light microscope (DMi8, Leica) using a 20x lens; for each time point, all microgels present on ten distinct images were analyzed. For doing so, for each microgel, the microgel diameter was measured at three different locations to calculate an average diameter for each sample. Moreover, two of such measured diameters had a pre‐set intersection angle of 90°, which allowed for calculating the aspect ratio of each microgel using a python program.

### 4.14 Enzymatic Degradation

To investigate the enzymatic degradation behavior of the microgels, mAlg‐mMUC gels with a biopolymer mixture ratio of 3:1 were prepared as described above. After cleaning the microgels using PFO, the samples were centrifuged and resuspended in either a 20 mg mL^−1^ Trypsin solution (Trypsin from porcine pancreas, 1000–2000 BAEE units mg^−1^, Sigma‐Aldrich) or in a 2 mg mL^−1^ Lyase (Alginate Lyase ≥ 10,000 units g^−1^ solid, Sigma‐Aldrich) solution prepared in DMEM. Then, the microgels were incubated in a 96‐well plate (Nest Biotechnology Co. Ltd., Wuxi, China) at room temperature and imaged using a fluorescence microscope (DMi8, Leica) equipped with a 20× objective (HC PL FLUOTAR L 20×/0.40 DRY) and a digital camera (Orca Flash 4.0 C11440, Hamamatsu) using TXR filter cubes (*E*_x_ = 540–580, DC =  585; *E*_m_ = 592–668, Leica).

### 4.15 Indentation Tests

To assess the mechanical properties of the microgels, indentation measurements were performed using a microscope‐compatible stage‐top nanoindenter (Chiaro, Optics11 Life, Amsterdam, Netherlands) employing an adapted protocol by Harder et al. [58]. A measurement probe with a 10.5 µm tip radius and a stiffness of 0.022 N m^−1^ was used. Indentation was carried out with a maximum load of 0.01, 0.05, or 0.1 µN (depending on the sample stiffness) with a piezo speed of 10 µm s^−1^.

Prior to indentation, the microgels were immobilized via a dopamine hydrochloride (dopamine, Sigma‐Aldrich) coating. To do so, dopamine was dissolved 0.4% (w/v) in tris(hydroxymethyl)‐aminomethane buffer (TRIS, Sigma‐Aldrich, 50 mM, pH 8.5) and added into the middle of the wells of a 24 well plate (Nest Biotechnology Co. Ltd.). For indentation measurements, the wells were filled with 3 mL ddH_2_O and 10 µL of sample solution. Measurements were performed at room temperature and were analyzed by fitting the obtained force curves with a Hertzian contact model implemented in the Optics11 Software (Prova v.0.9.0 Dev) to calculate the elastic modulus from each measurement with a set maximum indentation depth of 1500 nm. The Poisson's ratio was set to 0.5. For each sample type, a minimum of ten distinct microgels was evaluated; here, several indentations were performed on each microgel resulting in an average value of stiffness for each hydrogel. It is important to note that especially the de‐cross‐linked samples (after the addition of the chelator) show values which are at the detection limit of the indenter. Here, only fewer repetitions on each sample were possible, as the software could not fit the detected signal anymore and the application of force seemed to strongly affect the microgel (even leading to damage in some cases).

#### 4.15.1 Ionic Cross‐Linking

To demonstrate the option of increasing the stiffness of the microgels post‐production, additional ionic cross‐linking was carried out. Therefore, 100 µL of a 0.5 M CaCl_2_ solution was added into the well containing the microgels tested. Then, the indentation setting was adapted to a maximum load of 0.1 µN, as a higher stiffness of the microgels and thus a higher force necessary to achieve sufficient depth of indentation was expected.

#### 4.15.2 Chelator Induced Removal of Ionic Cross‐Links

To reverse the cross‐linking of alginate chains induced by Ca^2+^ ions, a chelator mix containing 0.5 M ethylenediaminetetraacetic acid (EDTA) and 0.25 M citrate buffer [34] (sodium citrate dihydrate and citric acid, VWR, Leuven, Belgium) was added to the microgel suspension. Owing to the high concentration of chelators in this mixture, adding a low volume of 4 µL was sufficient to induce rapid softening of the microgels. Please note that stiffness values below 100 Pa are not fully reliable, as this exceeds the sensitivity of the measurement setup used here.

#### 4.15.3 Poly‐L‐lysine Coating

To permanently increase the stiffness of different microgel formulations, a PLL (150–300 kDa, 0.01%, Sigma‐Aldrich) coating was carried out with slight adaptations. The samples were first transferred into a 15 mL tube and then centrifuged at 370 rcf for 5 min; afterwards, the supernatant was discarded. Then, 5 mL of 0.001% PLL solution were added, mixed with the sample, and the sample was centrifuged at 370 rcf for 5 min. The supernatant was discarded, and 2 mL of ddH_2_O were added, followed by centrifugation at 370 rcf for 5 min. This process was repeated one more time. The stiffness of these PLL‐coated microgel samples was then measured as described above. However, now, the maximum load was directly set to 0.1 µN and only reduced to 0.01 µN after treatment with the chelator solution.

### 4.16 Cytotoxicity Tests

To evaluate the (putative) cytotoxic potential of the microgels, in vitro cell culture tests were carried out. As a model cell line, NIH/3T3 mouse fibroblasts were selected and cultured in Dulbecco's eagle's high glucose medium (DMEM, Sigma‐Aldrich) supplemented with 1.0% (v/v) P/S and 10.0% (v/v) FBS in a 96 well plate (5000 cells per well, Nest Biotechnology Co. Ltd.) at 37°C and 5% CO_2_. Then, microgels (∼250 µL for each material composition; formulations as described above) were prepared as described above (including covalent cross‐linking via illumination with green light) and incubated with 2 mL of cell culture medium overnight to allow unreacted photoinitiator residues to leach out. On the next day, the samples were centrifuged at 600 rcf for 1 min to remove the microgels, and the supernatant was incubated with fibroblasts (200 µL per well), which were cultivated for another 24 h.

Additionally, to ensure that a sufficiently high amount of material is present when generating the leaching medium (this might not be the case when incubating microgels only), a second test was performed. Therefore, the relevant material compositions were prepared at the macroscale but directly cross‐linked via illumination with green light instead of fabricating microgels from them. Afterwards, these bulk gels (∼480 µL each) were incubated with medium overnight to generate a ‘leaching medium’ (by removing the supernatant after incubation), which was then added to cells seeded in a 96 well plate. Also here, the cells were incubated with this ‘leaching medium’ for 24 h.

In either case, the cells were washed with Dulbecco's phosphate buffered saline (DPBS, Sigma‐Aldrich) and stained with a live/dead staining containing 1 µM calcein and 2 µM ethidium homodimer‐1 (Invitrogen, Carlsbad, CA). As a negative control, cells were incubated with sterile methanol for 2 min before further treatment, and such methanol exposure killed all cells (data not shown).

Afterwards, the cells were imaged on a fluorescence microscope (Leica) equipped with a 20× objective (HC PL FLUOTAR L 20×/0.40 DRY) and a digital camera (Orca Flash 4.0 C11440, Hamamatsu) using FITC (*E*_x_ = 460–500, DC = 505; *E*_m_ = 512–542, Leica) and TXR filter cubes (*E*_x_ = 540–580, DC = 585; *E*_m_ = 592–668, Leica), respectively. The recorded images were analyzed using the software ImageJ (version 1.54 g). Each experiment was performed in *n* = 6 independent wells, and 3 microscope images were obtained from each well.

### 4.17 Biofouling Assay

To investigate the bacteria‐repellent properties of the mucin glycoprotein, a biofouling assay was performed with *E. coli* bacteria (*E. coli* TB205, mCherry labeled; TB205 was a gift from Tobias Bergmiller & Calin Guet (Addgene plasmid # 230034) [59]). For doing so, pure Alg and mAlg‐mMUC microgels (produced at mixture ratios of 1:1 and 3:1) were produced as described above. The microgels were then centrifuged, the water phase was replaced with a solution of planktonic bacteria (*E.coli* in LB medium (2.5%, X968.4) grown to an OD_600_ of ∼0.38 ± 0.02), and the mixture was incubated at 37°C under slight shaking for 7 days. Subsequently, the samples were centrifuged at 1 rcf for 5 min, and the supernatant was discarded. Fresh LB medium was added, the sample was slightly shaken, and the process was repeated (to remove unbound and weakly bound bacteria from the microgel solution). Afterwards, fresh LB medium was added, the samples were transferred to a 96 well plate (Nest Biotechnology Co. Ltd.) and they were imaged using a fluorescence microscope (DMi8, Leica) equipped with a 10× (HC PL FLUOTAR 10x/0.32 DRY) objective and a digital camera (Leica K8 CMOS) a DFT51010 filter cube (*E*_x_ = 391/32, 479/33, 554/24, 638/31, DC = 415, 500, 572, 660; *E*_m_ = 435/30, 519/25, 594/32, 695/58, Leica).

### 4.18 Statistics

Statistical tests were performed using the software OriginLab (Northampton, Massachusetts, USA). To test for normal data distribution, a Shapiro‐Wilk test was conducted. Then, a two sample Student's t‐test was performed in situations with normally distributed populations and similar variances whereas a Wilcoxon signed‐rank test was carried out when the variances were unequal. A Mann‐Whitney test was employed if the values were not normally distributed. In all cases, a *p*‐value of *p* ≤ 0.05 (corresponding to a confidence level of 95%) was chosen as a threshold for significance; significant differences were labelled with an asterisk.

## Author Contributions

**Jana T. Reh**, **Berna Özkale**, and **Oliver Lieleg** designed the experiments; **Leonard Funke** conducted the NMR measurements and assisted with the microindentation tests; all other experiments were conducted by **Jana T. Reh**. The manuscript was written by **Jana T. Reh** and **Oliver Lieleg** and critically revised by all authors.

## Conflicts of Interest

The authors declare no conflicts of interest.

## Supporting information




**Supporting File 1**: smll76505‐sup‐0001‐SuppMat.pdf.

**Supporting File 2**: smll76505‐sup‐0002‐VideoS1.mp4.

**Supporting File 3**: smll76505‐sup‐0003‐VideoS2.mp4.

## Data Availability

The data that support the findings of this study are available from the corresponding author upon reasonable request.
