# Supplementary material for: Composite Microgels with Dynamically Switchable Stiffness
Source: Small. 2026 Oct 30;22(63):e76505. doi: 10.1002/smll.76505 (PMC13755999; doi:10.1002/smll.76505)
Supplement: Supplementary file 1 [file SMLL-22-e76505-s001.pdf]

Supporting Information for

Composite Microgels with Dynamically Switchable Stiffness

Jana T. Reh[1,2,3], Leonard Funke[3,4,5], Berna Özkale[3,4,5], and Oliver Lieleg[1,2,3*]

[1] School of Engineering and Design, Department of Materials Engineering, Technical University of Munich, Boltzmannstraße 15, 85748 Garching, Germany

[2] Center for Protein Assemblies (CPA), Technical University of Munich, Ernst-Otto-Fischer Straße 8, 85748, Garching, Germany

[3] Munich Institute of Biomedical Engineering, Technical University of Munich, Boltzmannstraße 11, 85748 Garching, Germany

[4] Microrobotic Bioengineering Lab (MRBL), Department of Electrical Engineering, School of Computation, Information and Technology, Technical University of Munich, Hans-Piloty-Straße 1, 85748, Garching, Germany

[5] Munich Institute of Robotics and Machine Intelligence, Technical University of Munich, Georg-Brauchle-Ring 60, 80992 Munich, Germany.

*: corresponding author

Center for Protein Assemblies (CPA), Technical University of Munich,

Ernst-Otto-Fischer Str. 8, 85748 Garching, Germany

e-mail: oliver.lieleg@tum.de

keywords: alginate, mucin, cross-linking, indentation, mechanical properties

ORCID: **JR** (0009-0005-1462-8311); **LF** (0009-0003-4823-0059); **BÖ** (0000-0002-3016-9363); **OL** (0000-0002-6874-7456)

Section S1: Biopolymer distribution within the microgels

To visualize the distribution of the two biopolymer components throughout the fabricated microgels, fluorescently labeled biopolymer variants were used (see main text). Then, the fabricated microgels were imaged on a fluorescence microscope (see main text). For the microgel variants shown in **Figure S1**, the biopolymer solution was mixed only *via* vortexing prior to microgel fabrication.

The obtained images clearly show that a pure vortexing treatment resulted in poor homogeneity of the biopolymers as well as a large size variation of such prepared microgels.

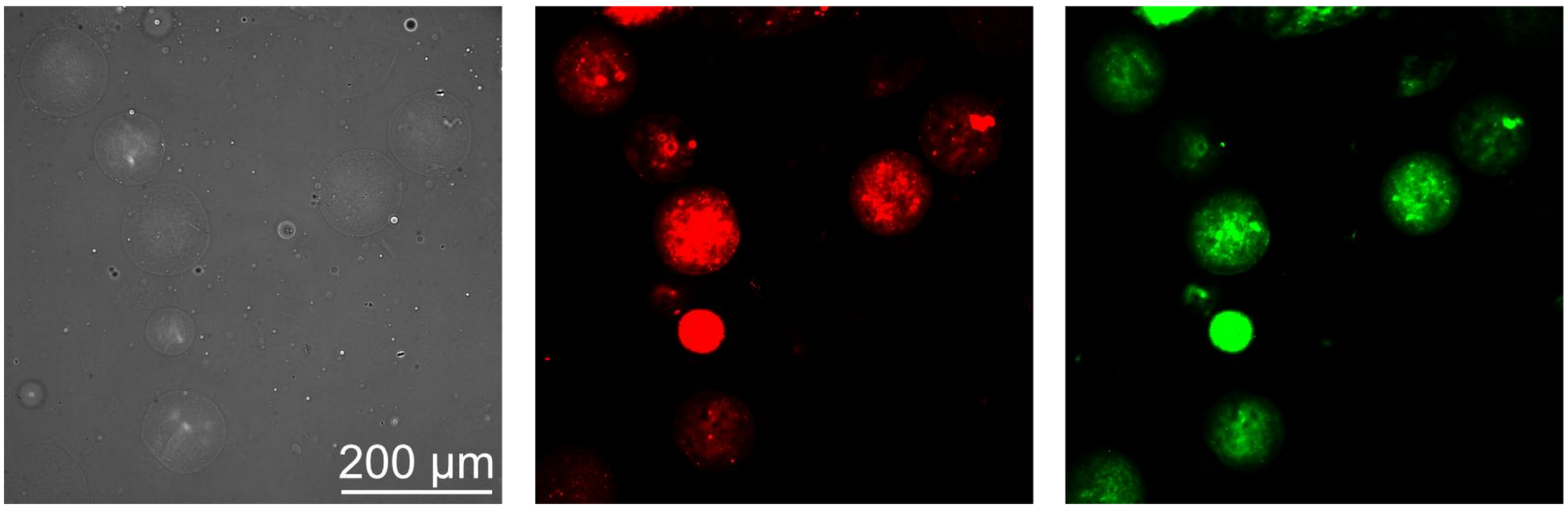

Figure S1: *Fluorescence microscopy images of microgels comprising a mAlg-mMUC mixture ratio of 1:1.* Prior to microgel fabrication, the biopolymer solution was mixed *via* vortexing only. The scale bar represents 200 µm and applies to all images in this figure.

Section S2: Additional light scattering results on the biopolymers

The biopolymers Alg and MUC as well as their functionalized variants (mAlg and mMUC) were characterized using dynamic light scattering. Here, the effect of tip sonication – in comparison to vortex treatment – on the hydrodynamic diameter was analyzed with a Litesizer 500 Zetasizer (Anton Paar). For these measurements, 0.1% (w/v) of a biopolymer was dissolved in ddH_2O and transferred into a four-clear-sided, disposable cuvette. The backscattering angle was determined by the device in an automated manner, and the hydrodynamic diameter was obtained *via* an intensity-average weighting model assuming a spherical particle geometry (**Figure S2a**,**b**). Zeta potential measurements were conducted as described in the main text.

We noted that, after tip sonication, the hydrodynamic diameter decreased for all biopolymer variants. However, for Alg and mAlg (**Figure S2a**) this effect was less pronounced than for MUC and mMUC (**Figure S2b**). This might result from the larger size of the biopolymer and its higher tendency to form aggregates compared to alginate. For both biopolymers, the decrease in hydrodynamic diameter was stronger for the methacrylated variants – yet those methacrylated variants both had larger initial hydrodynamic diameters than their unmodified counterparts. This suggested that methacrylation induced increased entanglements between the biopolymers resulting from the additional chemical moieties or an increased aggregation propensity brought about by the hydrophobic methacryl groups, and that tip sonication reverted this process. As all hydrodynamic diameters obtained after tip sonication were still in the range of $>10^3$ nm for Alg/mAlg and >200 nm for MUC/mMUC, biopolymer fragmentation is unlikely to have occurred. Moreover, the raw data obtained from the zeta potential measurements (**Figure S2c**) indicated that tip sonication benefited the samples: after tip sonication (lower row), the data distribution became more homogeneous in comparison to results obtained after vortexing treatment (upper row).

For the high MA variants of alginate and mucin (**Figure S2d-f**), the obtained data was consistent with the findings described above, *i.e.*, methacrylation seemed to induce aggregation/oligomerization, which was efficiently reverted by tip sonification (without affecting the zeta potential of the biopolymers).

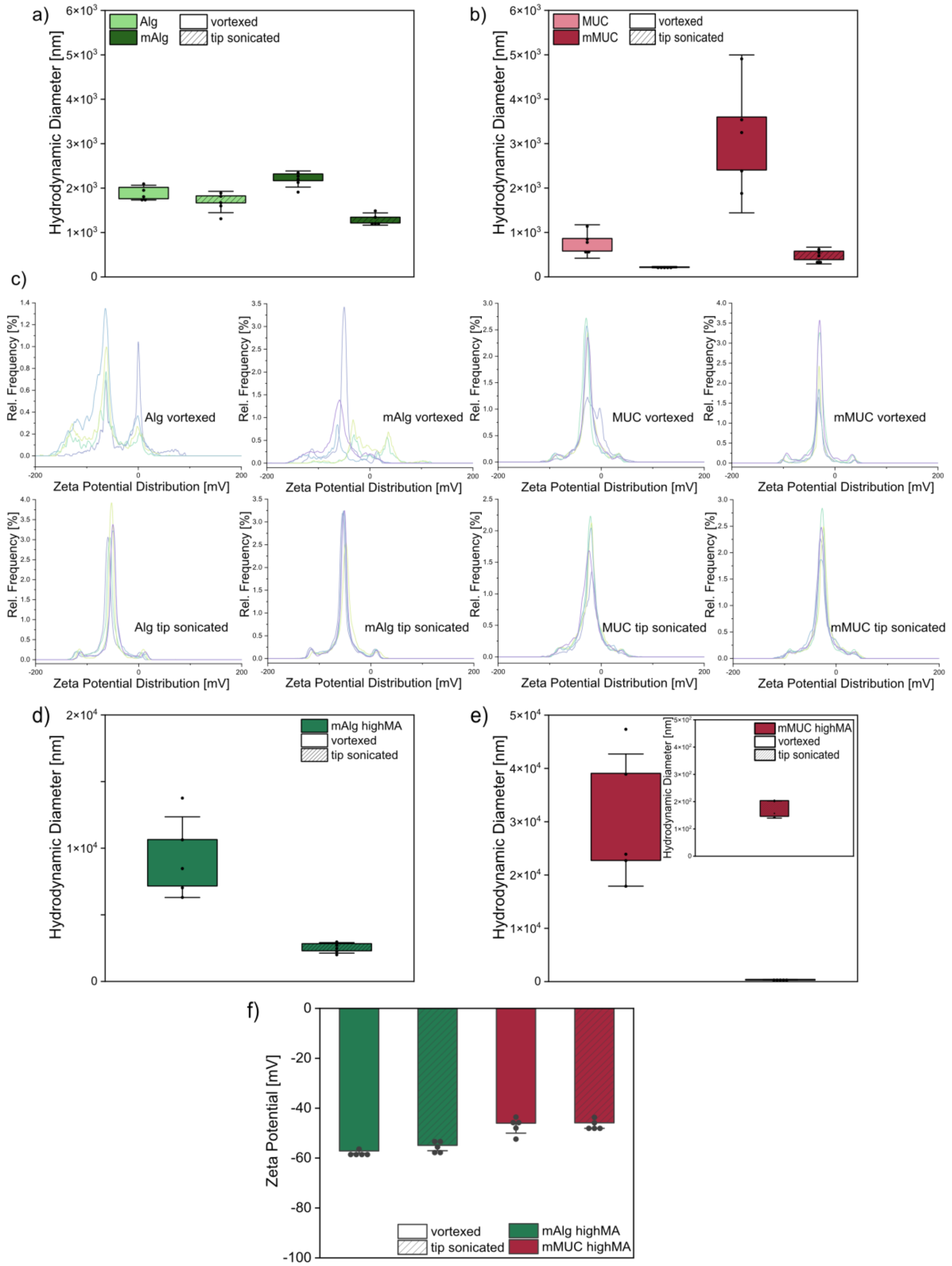

Figure S2: *Dynamic light scattering results obtained with different biopolymers.* Hydrodynamic diameter of (a) Alg and mAlg as well as (b) MUC and mMUC after vortexing and tip sonication treatment, respectively. *n* = 5. (c) Zeta potential distributions for Alg and MUC as well as their methacrylated variants after vortexing (upper row) and tip sonication (lower row), respectively. Hydrodynamic diameter of (d) mAlg high MA and (e) mMUC high MA after vortexing and tip sonication treatment, respectively. *n* = 5. (f) Zeta potential values of the mAlg high MA and mMUC high MA variants, both after vortexing or tip sonication treatment. *n* = 5. In all subfigures, error bars denote the standard deviation.

Section S3: Size and stiffness of additional microgel variants

As described in the main text, different ratios of mAlg to mMUC were investigated. Microgels with a material composition of mAlg to mMUC of 1:3 showed a low yield (**Figure S3a**) and thus were not considered further.

One strategy to induce the increase of the Young's modulus of alginate/mucin microgels was coating them with PLL. Here, not only the concentration of PLL in the incubation solution but also the duration of the incubation step (see **Figure S3b**) affected the final stiffness of the microgels.

When investigating the viscoelastic moduli of bulk gels produced from a higher total polymer concentration, we found a slight increase in stiffness (see **Figure S3c**). However, for the high MA variants of alginate and mucin, even stronger mechanical properties were obtained at the macroscale even at a total biopolymer concentration of 1% (w/v) (see **Figure S3c**).

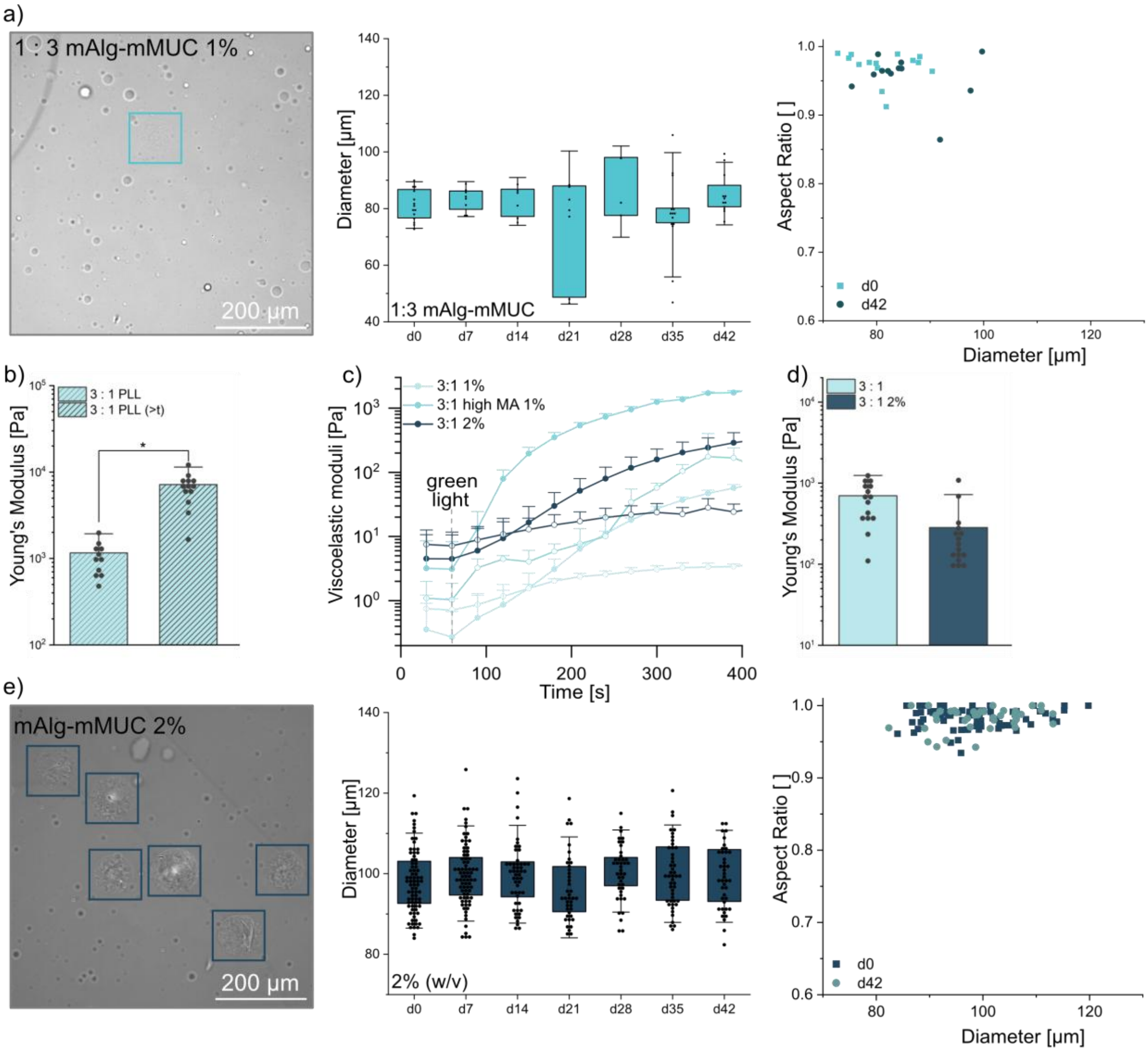

***Figure S3:** Morphology and mechanical properties of mixed mAlg-mMUC gels at the micro- or macroscale.* (a) Microgels were produced at an mAlg to mMUC ratio of 1:3 and a total polymer concentration of 1% (w/v). Afterwards, they were stored in ddH_2O at 4°C for 42 days. During this time, the average diameter of the microgels as well as their aspect ratio were determined. At each time point, 10 images were taken and all microgels in those images were analyzed. (b) Young's moduli of microgels as obtained *via* microindentation. Data for 3:1, 1% (w/v) PLL microgels was taken from the main text and compared to 3:1, 1% (w/v) PLL microgels where the incubation time in PLL was increased to 5 min. (c) Viscoelastic moduli *G'* (closed symbols) and *G''* (open symbols) of different macrogel samples (mAlg-mMUC 3:1, 2% (w/v) total biopolymer concentration; high MA mAlg-mMUC 3:1, 1% total biopolymer concentration) over time; illumination with green light was initiated after 1 min and induced gelation *via* covalent cross-linking. n = 3. (d) Young's moduli of microgels as obtained *via* microindentation. Data for 3:1, 1% (w/v) microgels was taken from the main text and compared to results obtained for 3:1, 2% (w/v) microgels. (e) Microgels were produced at an mAlg to mMUC ratio of 3:1 and a total biopolymer concentration of 2% (w/v). They were afterwards stored and analyzed as in (a). Error bars denote the standard deviation. Asterisks denote statistically significant differences based on a p-value of p = 0.05.

One alternative option to increase the stiffness of the generated microgels was to prepare them at a higher overall biopolymer concentration of 2% (w/v). When indenting 3:1 microgels produced from the high polymer concentration (2% (w/v)), the measured stiffness values were below the values obtained for 3:1 microgels produced from the lower polymer concentration of 1% (w/v) (see **Figure S3d**). This surprising outcome might have resulted from an uneven biopolymer distribution on the surface of the 2% (w/v) microgels (see in **Figure S4**).

Such microgels (prepared at a mAlg to mMuc ratio of 3:1) showed a constant size distribution in the range of 100 µm over 42 days of incubation at 4°C (see **Figure S3e**). In addition, the obtained aspect ratios indicated a well-spherical shape of the microgels.

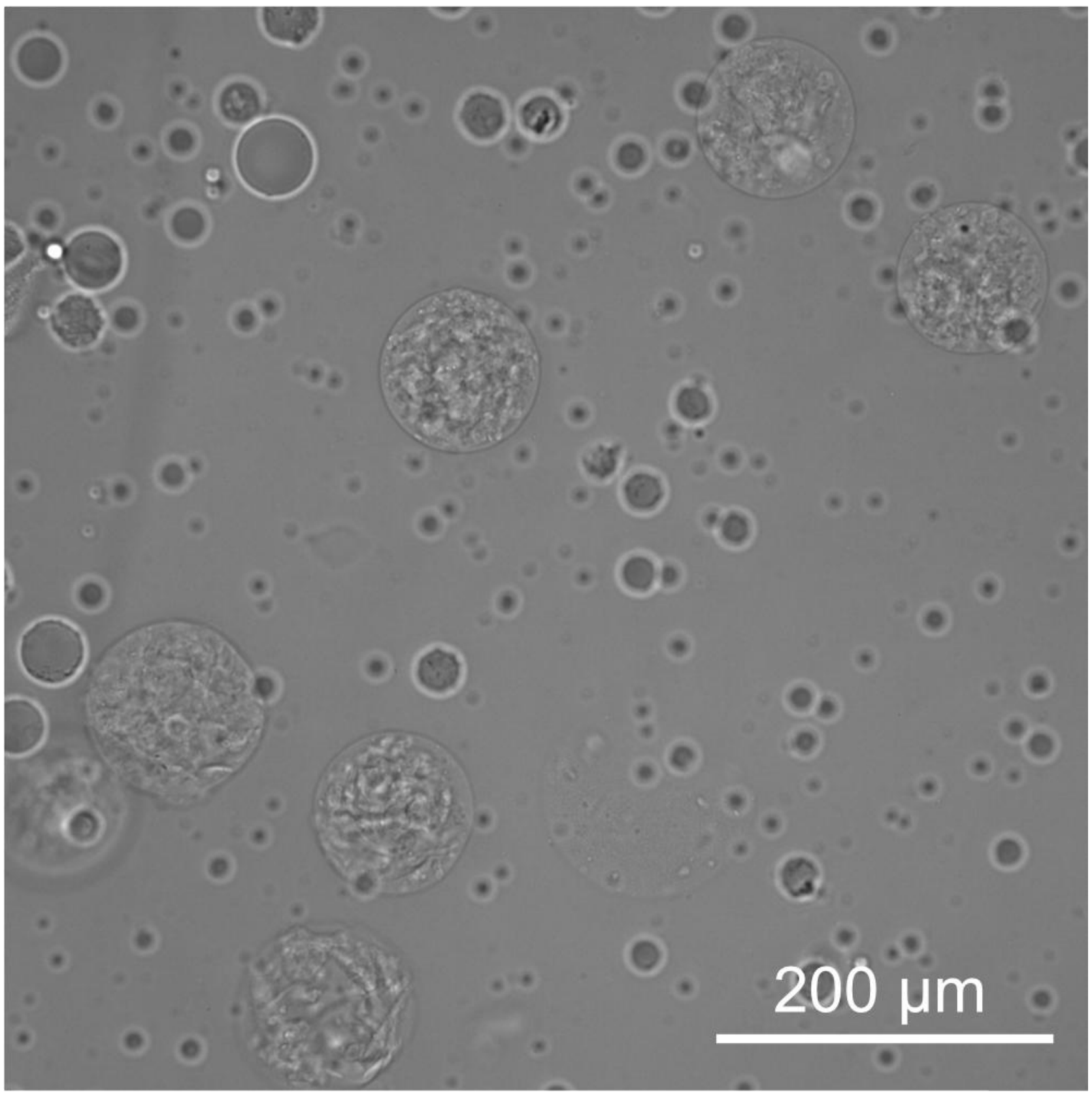

Figure S4: *Brightfield microscope image of mAlg-mMUC 3:1 microgels prepared at a total polymer concentration of 2% (w/v).* The scale bar indicates 200 µm.

Section S4: Enzymatic degradation

The enzymatic degradation behavior of the microparticles was investigated by adding Lyase or Trypsin. For both cases, microscopy images were taken over time to illustrate the degradation process of the microgels (see **Figure S5**). Please note that the images shown below were taken in the same respective sample but not at an identical position within the well.

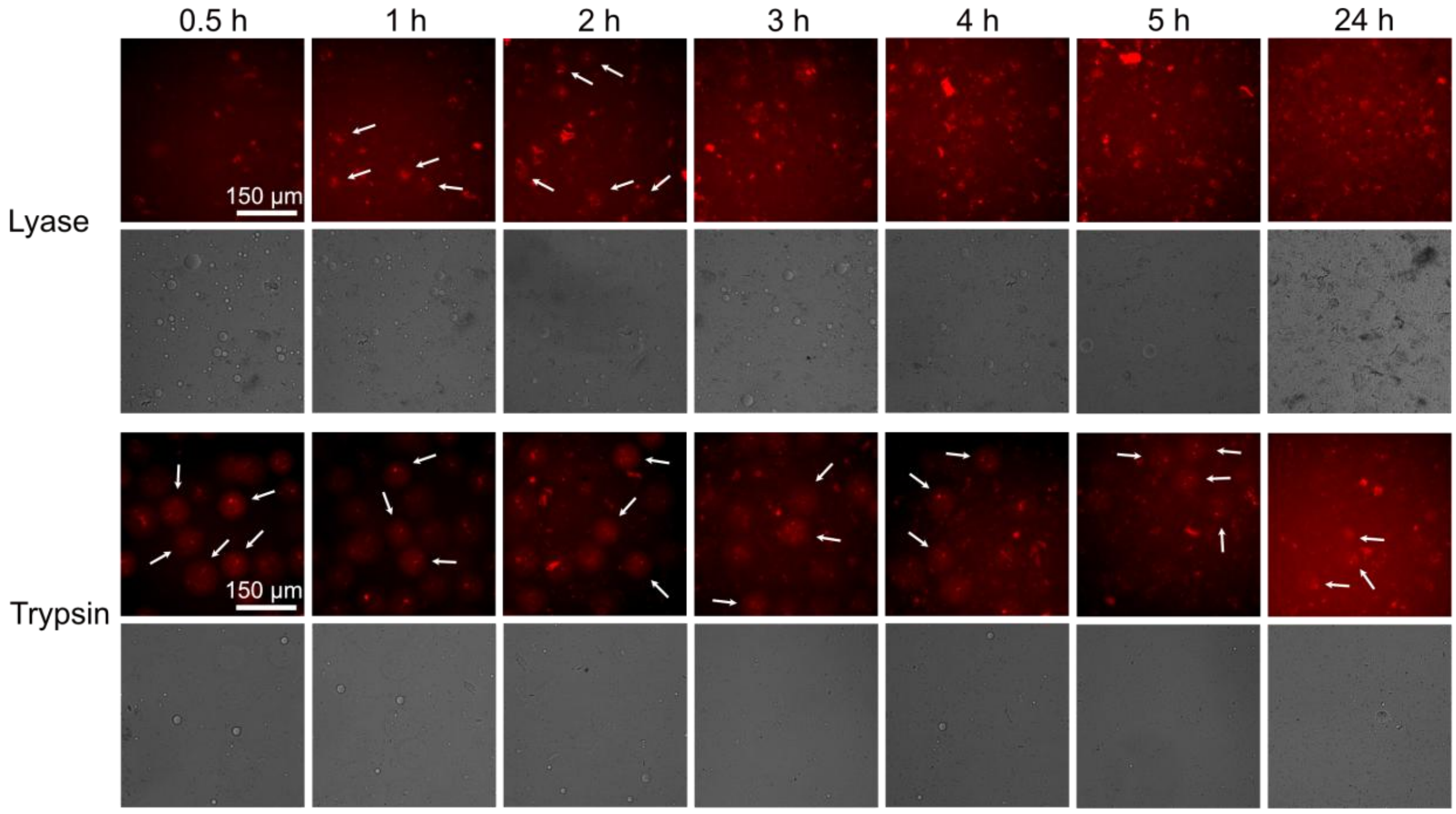

Figure S5: *Microscope images of mAlg-mMUC 3:1 microgels during an enzyme-driven degradation process.* The added arrows indicate exemplary microgels which are still considered intact. The scale bar indicates 150 µm and applies to all images in the figure.

Section S5: Nuclear magnetic resonance (^{1}H-NMR) spectroscopy

Nuclear magnetic resonance spectroscopy (^{1}H-NMR) was performed to determine the degree of the methacrylate functionalization of the polymers. For this purpose, 8 to 12 mg of sample were dissolved at 1% (w/v) in deuterium oxide (D_2O, Merck) overnight. The samples were subsequently freeze-dried and re-dissolved in D_2O. This process was repeated. A 10 mM stock solution of potassium phthalate (KHP, Merck) was used to add a final amount of 0.909 mM KHP to the D_2O solution, which was then mixed for 3 h.

Then, ^{1}H-NMR (Bruker EVO 500 MHz running TopSpin 4.5.0 software) was carried out. For alginate and its functionalized variants, spectra were obtained with 16 scans at

340 K. For mucin, spectra were obtained with 16 scans at 340 K but with water repression. When carrying out NMR measurements, KHP served as a control, whose peaks are known. In each spectrum, the corresponding peaks can be found at ~7.5 ppm and ~7.7 ppm. Successful methacrylation should result in the occurrence of methacrylate peaks around ~6.1 ppm and ~6.5 ppm.

The spectra obtained for unmodified and modified alginate are shown in **Figure S6**.

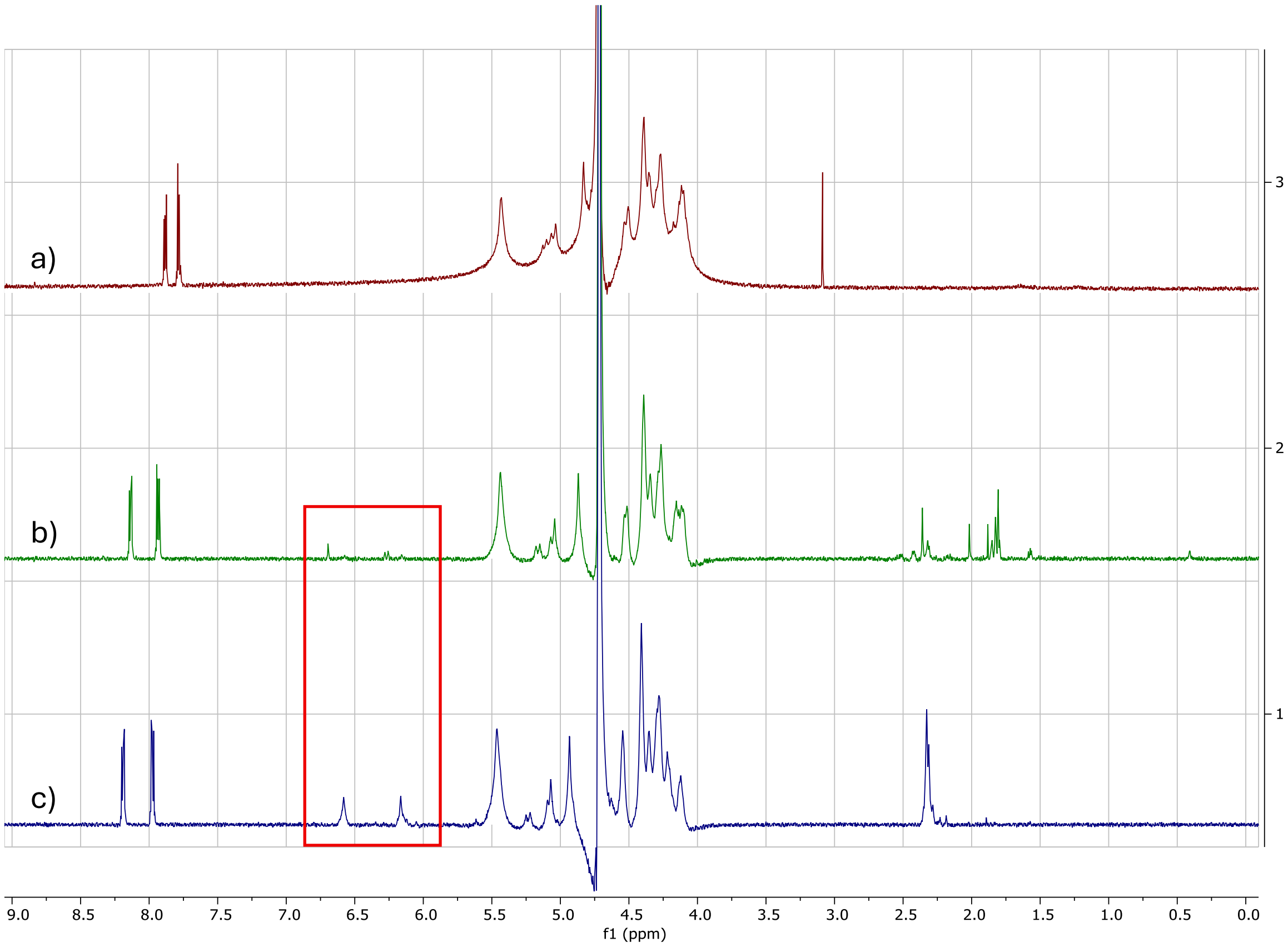

Figure S6: ^{1}H-NMR spectrum of (a) unmodified alginate, (b) mAlg, and (c) mAlg high MA.

For mAlg (green curve in **Figure S6b**), the characteristic peaks for methacrylate can be found. Those peaks were used to quantify the degree of methacrylation.

The improved, high MA version of alginate also shows the characteristic methacrylate peaks (blue curve in **Figure S6c**). Here, the peaks are more pronounced than in the mAlg spectrum, which is an indication of improved functionalization.

The success of the functionalization was then determined by calculating the relative molar ratio (MR) as well as the degree of substitution (DS).

Following a method by Chou *et al.* [1]), we obtained a MR of ~1.2% for mAlg and of ~21.3% for mAlg high MA. This result agrees with the improved mechanical properties observed for the mAlg high MA variant (see main text). For these calculations, a control peak from the alginate spectrum was considered.

When conducting the calculation according to Holzgrabe [2] (using the KHP control peak for the calculations), we obtained a DS ~1.3 for mAlg and DS of ~23.9 for mAlg high MA.

The spectra obtained for different mucin variants are shown in **Figure S7**.

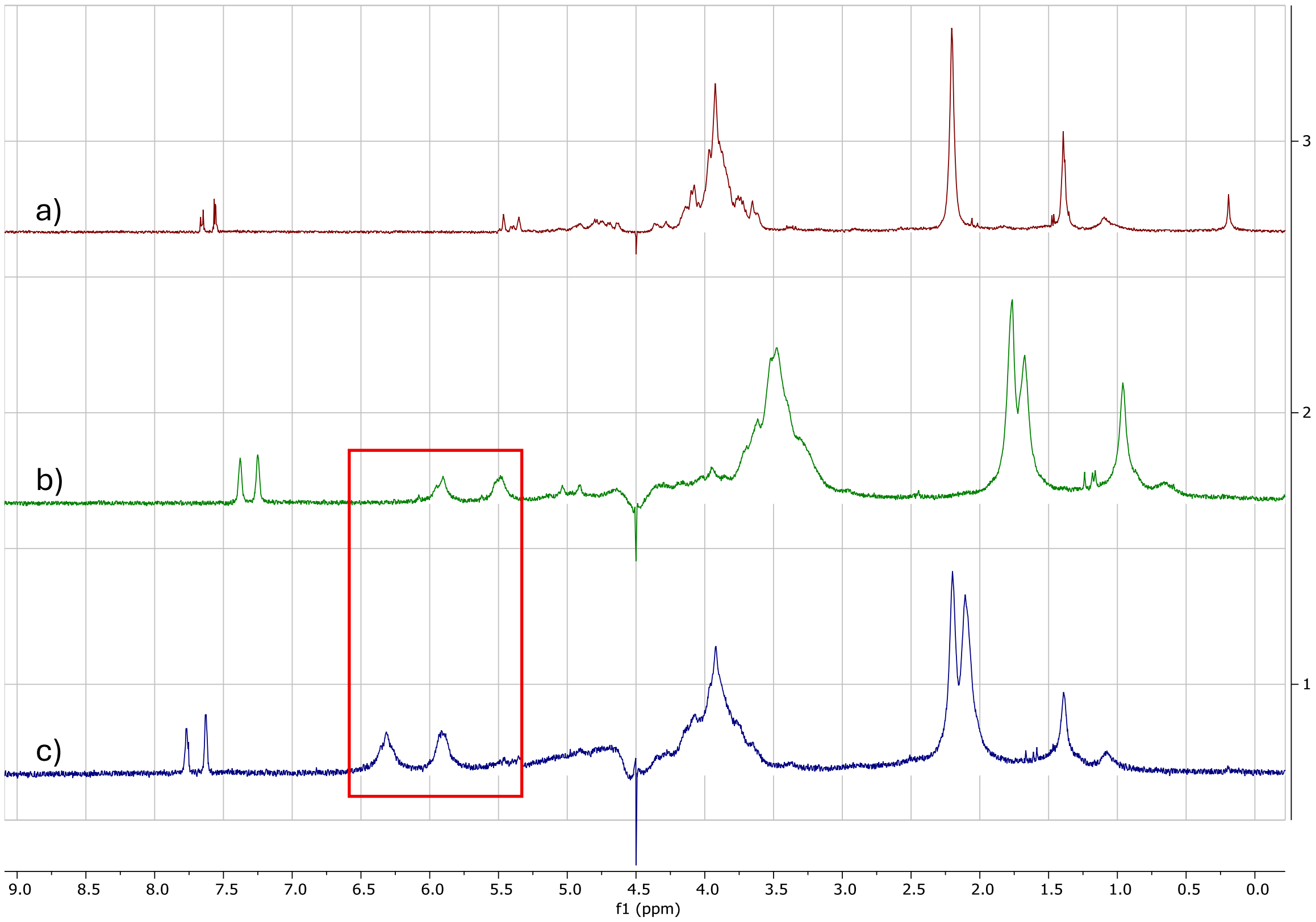

Figure S7: ^{1}H-NMR spectrum of (a) unmodified MUC5AC, (b) mMUC, and (c) mMUC high MA.

For mMUC, successful methacrylation is verified by the occurrence of the characteristic methacrylate peaks at ~6.1 ppm as well as at ~6.5 ppm (**Figure S7b**).

Also for mMUC high MA, the characteristic methacrylate peaks are clearly visible (**Figure S7c**).

For mMUC and mMUC high MA, the DS was calculated to be ~2k and ~4k respectively, assuming a molecular weight of 9 MDa [3] and considering the KHP peak. The MR could not be calculated due to the lack of known characteristic peaks in the spectrum of MUC5AC. In fact, as a result of the complex structure of MUC5AC, the obtained ^{1}H-NMR spectra show a variety of peaks, which potentially overlap and thus affect the determination of the MR.

Thus, to support the findings from NMR, additional absorbance measurements were conducted using a UV-Vis spectrometer (Varioskan LUX multimode microplate reader, Thermo Fisher Scientific, Waltham, USA) (see **Figure S8**). For those measurements, 0.01% (w/v) solutions of MUC, mMUC, mMUC high MA, Alg, mAlg, as well as mAlg high MA were prepared in ddH_2O. The absorbance spectra of the unmodified biopolymers MUC and Alg were set as blanks. Then, all methacrylated samples showed characteristic peaks between 210 nm and 220 nm [4], which is in accordance with the literature. Moreover, this methacrylate peak was clearly more pronounced for the high MA versions of both biopolymers.

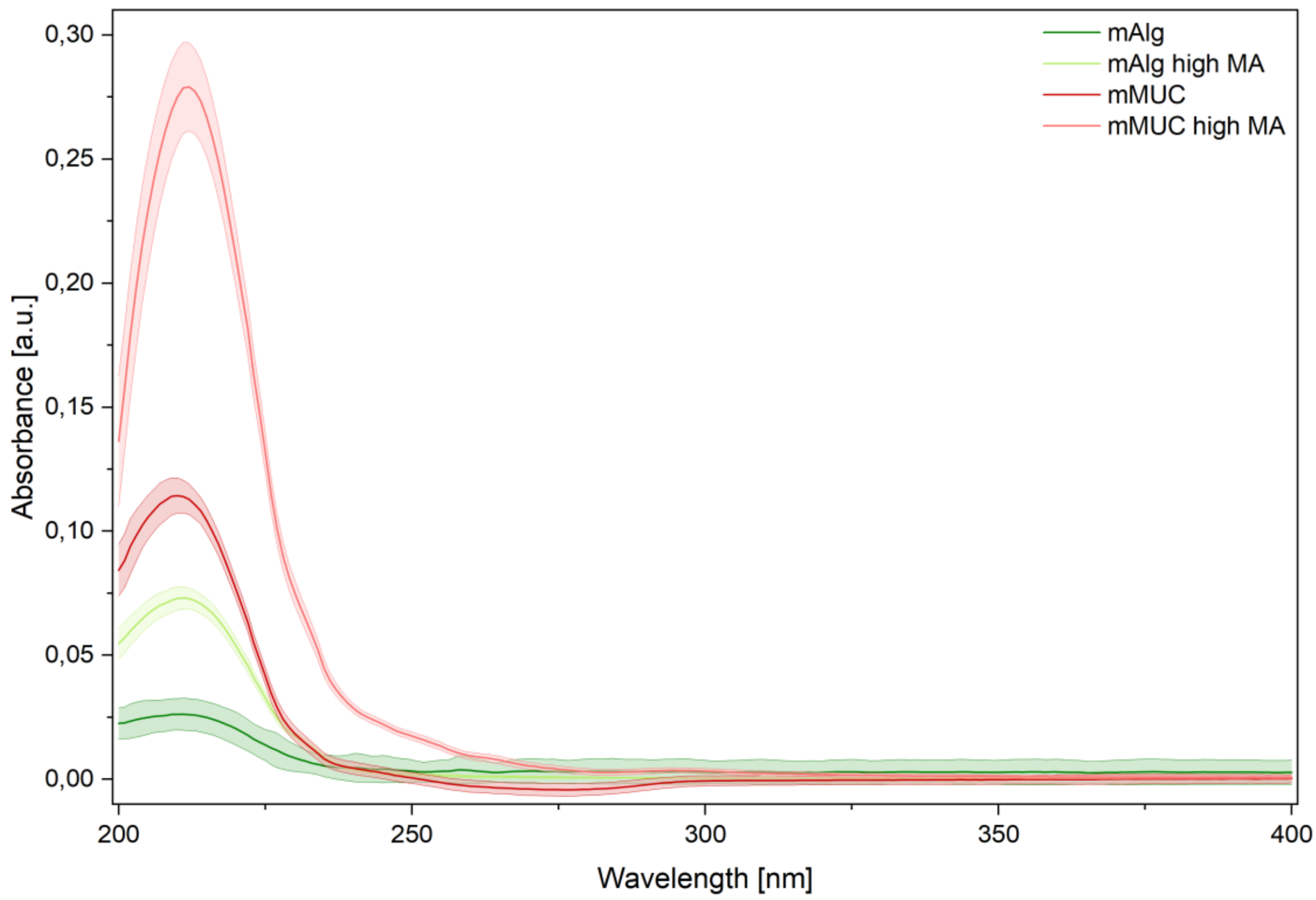

Figure S8: Absorbance spectra of mMUC, mMUC high MA, mAlg, and mAlg high MA. The graph shows the average of $n = 3$ measurements; shaded areas denote the standard deviation.

Section S6: Cyclic switching of the microgel stiffness

Adjusting the stiffness of the microgels can be performed in a cyclic manner as exemplarily shown for an alginate/mucin microgel created from a 3:1 mixture of those biopolymers in **Figure S9**.

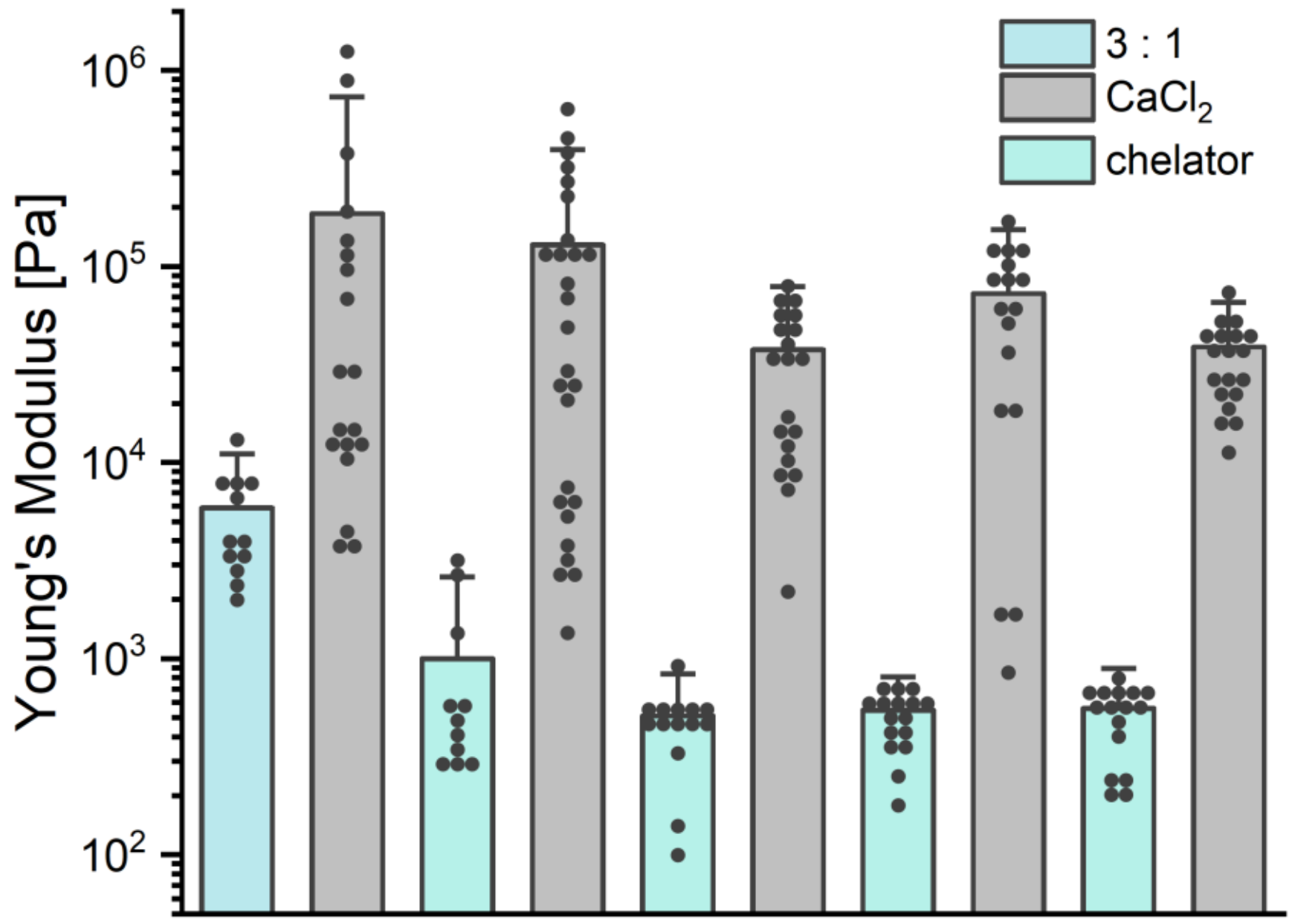

Figure S9: *Repeated indentation measurements of mAlg-mMUC 3:1*. The alternating addition of the $CaCl_2$ solution as well as the chelator mixture was carried out over several cycles. Bars denote the average, error bars denote the standard deviation.

The corresponding indentation measurement was carried out in a well of a 48 well-plate which had previously been coated with dopamine (see the main text for details). For triggering a cyclic change in the microgel stiffness, the $CaCl_2$ solution as well as the chelator mixture were added directly into the well in a stepwise manner. The indentation measurements were carried out directly after cross-linker/chelator addition using a softer indenting tip suitable for detecting especially the softer microgels. For the chelator mixture, 4 µL of the stock solution were added to the well during each step. For the $CaCl_2$ solution, different volumes (100 µL, 20 µL, 5 µL, 10 µL and 5 µL) of a 0.5 M stock solution were added. Here, one data point shown in the graph corresponds to a distinct measurement point obtained on a microgel. Per condition, about 11-27 different microgels were analyzed.

Section S7: Additional cytocompatibility and anti-biofouling tests

The cell experiments described in the main text were also performed for microgels produced from the high MA biopolymer variants as well as for microgels generated from a total biopolymer concentration of 2% (w/v). The corresponding results are compiled in **Figure S10a-c**. We found that neither of those additional microgel variants induced cytotoxic effects but showed similarly good cell viability as the control.

In another set of cytocompatibility tests, 50 µL of a microgel solution (~250 µL for each material composition in ~0.8 mL of cell culture medium) were directly added to the cells into the wells and incubated overnight. However, in those direct exposure tests, the microgels interfered with the staining process as they absorbed both dyes, which rendered an image quantification impossible. Thus, such obtained images were only interpreted based on the appearing cell morphology. However, also those tests did not return any signs of cytotoxic behavior for cells that were in direct contact with microgels.

Figure S10d shows exemplary images of such situations, where microgels were directly incubated on top of the seeded NIH/3T3 cell layer. Since the microgels and the cells were located in different focus planes, in the brightfield images, the focus was set to fit the microgel position; in contrast, in the fluorescence images, the focus was set to fit the z-level of the stained cells. Please note that both types of images were always taken at the same x/y position, and the microgels were marked with white borders for clarity. After application of the live/dead stain, no dead (= red stained) cells were visible in either merged image. This indicated that, also upon direct contact with the cells, the microgels did not exert any cytotoxic effects. However, as the microgels somewhat interfered with the staining process (since they absorb either dye), these images were not quantified.

The anti-biofouling characteristics of the microgels were also investigated for the comparably higher concentration of mMUC present in mAlg-mMUC 1:1 microgels (see **Figure S10e**). Also for this microgel composition, mMUC conveys anti-biofouling properties to the microgels.

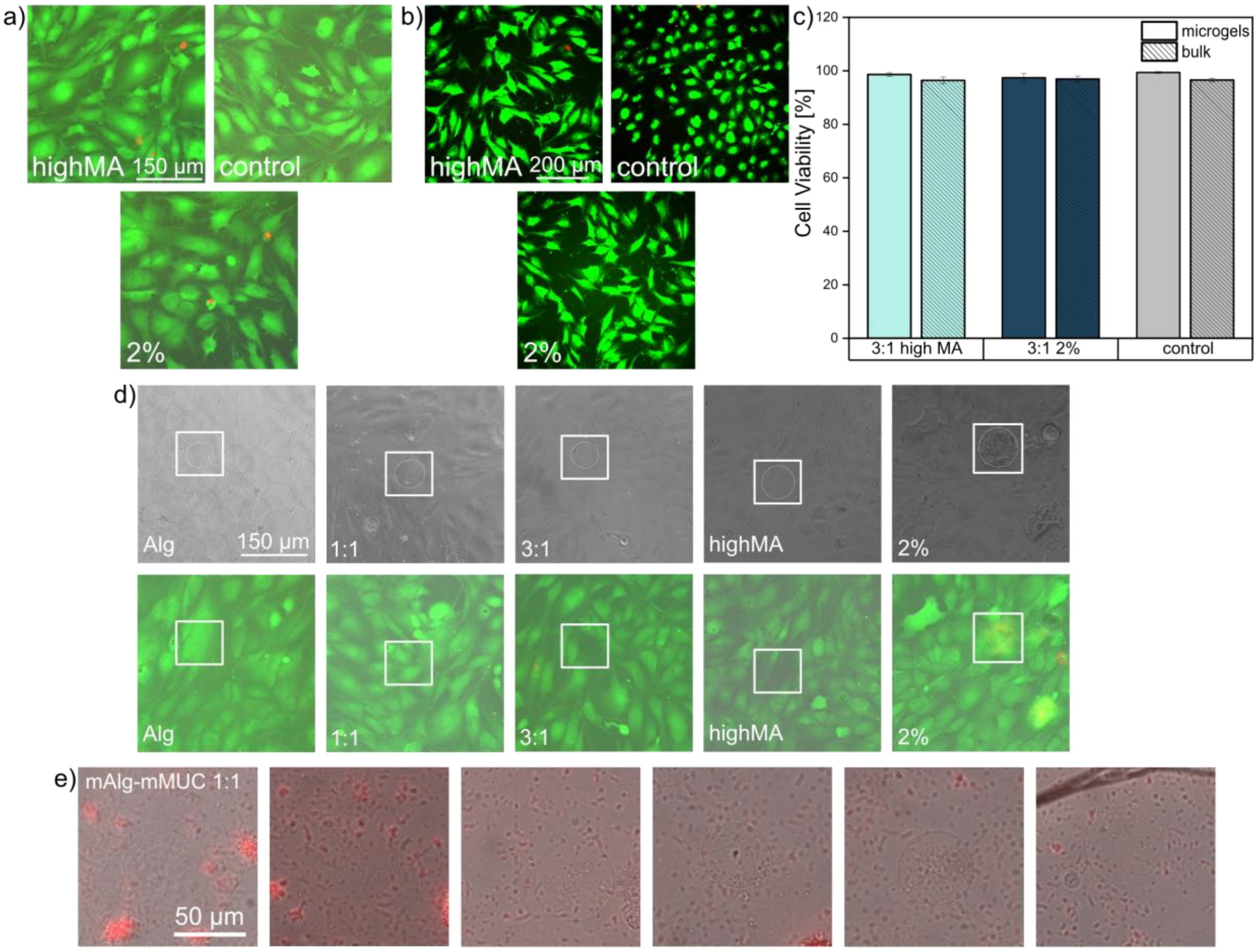

Figure S10: *Cytocompatibility analysis of mixed alginate/mucin gels*. Exemplary microscope images showing results of a live/dead staining test conducted with (a) microgels and (b) bulk gels, each prepared at a biopolymer mixture ratio of 3:1. Samples with a higher total biopolymer concentration of 2% as well as samples produced from high MA variants of the biopolymers (total biopolymer concentration of 1%) are analyzed. The scale bars represent 150 µm and 200 µm, respectively and apply to all images in the corresponding subfigure. (c) Cell viability values as calculated from the images shown in (a) and (b) as well as similar ones. Cells incubated with medium only were used as a control group. (d) Exemplary images depicting the results from a live/dead staining test after direct incubation of cells with microgels. The upper row depicts bright field images, the lower row merged images (bright field and fluorescence). Pure alginate microgels (Alg) as well as mixed alginate/mucin microgels prepared at a ratio of 1:1 and 3:1 are (total biopolymer concentration: 1 % (w/v) are compared to 1:1 microgels prepared from the high MA variants of the biopolymers and 1:1 microgels prepared at a higher biopolymer concentration of 2 % (w/v). Since the microgels are sitting on top of the cell layer, the focus had to be adjusted when imaging the microgels and cells, respectively. In the fluorescence images, the position of the (out-of-focus) microgels is marked with a box. The scale represents 150 µm and applies to all images in this subfigure. (e) Exemplary microscope images of a biofouling experiment investigating the adhesion of bacteria to mAlg-mMUC 1:1 microgels. The scale bar represents 50 µm and applies to all images in this subfigure.

1. Chou, A.I., S.O. Akintoye, and S.B. Nicoll, *Photo-crosslinked alginate hydrogels support enhanced matrix accumulation by nucleus pulposus cells in vivo*. Osteoarthritis Cartilage, 2009. **17**(10): p. 1377-84.
2. Holzgrabe, U., *Quantitative NMR spectroscopy in pharmaceutical applications*. Progress in Nuclear Magnetic Resonance Spectroscopy, 2010. **57**(2): p. 229-40.
3. Carpenter, J., et al., *Assembly and organization of the N-terminal region of mucin MUC5AC: Indications for structural and functional distinction from MUC5B*. *PNAS*, **2021**. **118**(39).
4. Rad, N., et al., *Routine Direct Quantification of Methacrylic Groups per Gelatin Unit in Gelatin Methacryloyl Using Ultraviolet Spectroscopy*. Biomacromolecules, 2026. **27**(4): p. 2572-2582.
